# The success rate of processed predicted models in molecular replacement: implications for experimental phasing in the *AlphaFold* era

**DOI:** 10.1107/S2059798324009380

**Published:** 2024-10-03

**Authors:** Ronan M. Keegan, Adam J. Simpkin, Daniel J. Rigden

**Affiliations:** a Institute of Systems, Molecular and Integrative Biology University of Liverpool LiverpoolL69 7ZB United Kingdom; b UKRI–STFC Rutherford Appleton Laboratory Research Complex at Harwell DidcotOX11 0FA United Kingdom; ESRF, France

**Keywords:** molecular replacement, *AlphaFold*2, computational methods, experimental phasing

## Abstract

The availability of highly accurate protein structure predictions from *AlphaFold*2 (AF2) and similar tools has hugely expanded the applicability of molecular replacement (MR) for crystal structure solution. Many structures can be solved routinely using raw models, structures processed to remove unreliable parts or models split into distinct structural units. There is therefore an open question around how many and which cases still require experimental phasing methods such as single-wavelength anomalous diffraction (SAD). Here, this question is addressed using a large set of PDB depositions that were solved by SAD. A large majority (87%) could be solved using unedited or minimally edited AF2 predictions. A further 18 (4%) yield straightforwardly to MR after splitting of the AF2 prediction using *Slice’N’Dice*, although different splitting methods succeeded on slightly different sets of cases. It is also found that further unique targets can be solved by alternative modelling approaches such as *ESMFold* (four cases), alternative MR approaches such as *ARCIMBOLDO* and *AMPLE* (two cases each), and multimeric model building with *AlphaFold-Multimer* or *UniFold* (three cases). Ultimately, only 12 cases, or 3% of the SAD-phased set, did not yield to any form of MR tested here, offering valuable hints as to the number and the characteristics of cases where experimental phasing remains essential for macromolecular structure solution.

## Abstract 2

A large set of SAD-phased PDB depositions were tested for solubility by molecular replacement using *AlphaFold*2 and other approaches. The 3% of cases that were not solved help to show where experimental phasing efforts could be focused in the future.

## 1. Introduction

For many years, molecular replacement (MR) has been the predominant mode of solution of the phase problem in macromolecular structure determination, and its popularity has recently increased. MR depends on identifying a search model that is sufficiently similar to at least part of the new crystal structure, allowing its computational placement in the asymmetric unit of the new structure. An initial set of calculated phases can then be determined, allowing the production of initial electron-density maps which may then ultimately allow structure tracing and refinement. Conventionally, the search model has typically been derived from the experimental structure of a quite closely related homologous protein, but the area has also been fertilized by structural bioinformatics. This latter field has encompassed the testing of small substructures that are identifiable by secondary-structure prediction (Rodríguez *et al.*, 2009 ▸), diverse approaches for the definition of useful substructures in more distantly related homologues (Sammito *et al.*, 2014 ▸; Rigden *et al.*, 2018 ▸) and the exploitation of successive waves of *ab initio* structure-modelling methods (Qian *et al.*, 2007 ▸; Bibby *et al.*, 2012 ▸; Simkovic *et al.*, 2016 ▸; Simpkin *et al.*, 2019 ▸).

The latest deep-learning-based structure-prediction methods, starting with *AlphaFold*2 (AF2; Jumper *et al.*, 2021 ▸), have had a profound effect on MR. It was immediately recognized at and after the CASP14 structure-prediction experiment (Millán *et al.*, 2021 ▸; Pereira *et al.*, 2021 ▸; McCoy *et al.*, 2022 ▸) that their unprecedented accuracy made many targets that would previously have been difficult or intractable, principally those of significantly novel folds, readily soluble by MR. Often an unmodified AF2 model would succeed as a search model, but the importance, in some cases, of removing less confident (often disordered) portions of models and/or splitting multi-domain structure predictions into individual domains was quickly recognized (Oeffner *et al.*, 2022 ▸; Simpkin *et al.*, 2022 ▸). A study of 215 recent PDB (Berman *et al.*, 2003 ▸) depositions that had been solved by single-wavelength anomalous diffraction (SAD) (and which were hence assumed to comprise structures not readily soluble by MR) found that 87% were easily solved using AF2 models, with only seven ultimately failing in MR (Terwilliger *et al.*, 2023 ▸). Such results have encouraged the integration of AF2 into structure-solution pipelines (Krissinel *et al.*, 2022 ▸; Poon *et al.*, 2024 ▸; Simpkin, Caballero *et al.*, 2023 ▸).

Here, we address the extent to which search models from AF2 and other approaches can solve recent PDB depositions that had been solved by single-wavelength anomalous diffraction (SAD). The set of 408 cases, deposited between 27 July 2022 and 4 October 2023, is much larger than those tested in previous work but, it should be remembered, represents only a minority of PDB entries: in the same period 10 084 deposited structures were listed as being determined using MR. Crucially, as well as testing the performance of different AF2 variants [the original DeepMind version (Jumper *et al.*, 2021 ▸) and *ColabFold* (Mirdita *et al.*, 2022 ▸)], we quantify the importance of (automated) domain splitting, as well as illustrating the key value in difficult cases both of other software [such as *ESMFold* (Lin *et al.*, 2023 ▸) and *ARCIMBOLDO* (Rodríguez *et al.*, 2009 ▸)] and of alternative approaches (for example multimeric search-model building). All in all, just 3% of the SAD test-set structures were not solved (or 0.1% of the 10 492 total depositions), providing a new picture of the size and the characteristics of recent PDB depositions for which experimental phasing was considered likely to be essential for structure solution. We find that targets with few homologues in the database are challenging, as expected, and that proteins with predominantly α secondary structure, especially coiled coils, are also over-represented in difficult cases. While experimental phasing is widely used for other purposes (El Omari *et al.*, 2023 ▸), for example localization of ions, these results help to define the future targets which should be prioritized for this typically more expensive and laborious mode of structure solution.

## 2. Methods

### 2.1. Selection of target sequences

The targets selected were PDB structures that had been solved by single-wavelength anomalous diffraction (SAD) and deposited between 27 July 2022 and 4 October 2023. This yielded 408 cases, two of which (PDB entries 8ec9 and 8cuf) contained peptides with multiple modified or unnatural amino acids and so were excluded (see the supporting information for a list of PDB codes).

### 2.2. Modelling and characterization of target sequences

Target sequences were modelled using DeepMind *AlphaFold*2 (here called AF2; Jumper *et al.*, 2021 ▸) and *ColabFold* (CF; Mirdita *et al.*, 2022 ▸). These methods employ the same trained network but differ in other aspects, principally in the mode of generation of the multiple sequence alignment (MSA), a key input. DeepMind AF2 uses *HHblits* (Remmert *et al.*, 2012 ▸) and *jackhmmer* (Johnson *et al.*, 2010 ▸) to search the Big Fantastic Database (BFD) and the Uniclust30 (Mirdita *et al.*, 2017 ▸), UniRef90 (Suzek *et al.*, 2015 ▸) and MGnify (Richardson *et al.*, 2017 ▸) databases, while *ColabFold* uses the *MMseqs*2 application programming interface (Steinegger & Söding, 2017 ▸), allowing a rapid search of metagenomic databases. By default, AF2 uses structural templates (homologous structures identified in the PDB), whereas CF does not. Where single-molecule AF2 and CF predictions failed to yield a solution, *ESMFold* models (Lin *et al.*, 2023 ▸) were generated. Multimeric search models were also generated for these cases using *AlphaFold-Multimer* (Evans *et al.*, 2021 ▸) or *Uni-Fold Symmetry* (Li *et al.*, 2022 ▸).

Alignment depth was expressed as Neff, a measure of the effective number of sequences for MSAs (Wu *et al.*, 2020 ▸; Jumper *et al.*, 2021 ▸), using an 80% sequence-identity threshold and normalized by target length, hence Neff/length.

Targets were classified using the secondary-structure information in the deposited PDB structures, defining them as all-α (100% of regular secondary structure was α-helix), mostly α (>65% α), mixed, mostly β (>65%) or all-β (100% of regular secondary structure was β-sheet).

*Socket*2 (Kumar & Woolfson, 2021 ▸) was used to evaluate whether targets contained coiled-coil domains within them, as it has been noted that AF2 struggles to accurately predict these domains (van Breugel *et al.*, 2022 ▸).

### 2.3. Processing of structure predictions into search models

AF2 predictions are accompanied by residue-level confidence estimates expressed as predicted local difference distance test (pLDDT) values on a scale of 0 to 100, where values of 70 or higher are considered as moderate-to-high confidence (Jumper *et al.*, 2021 ▸). The AF2 and CF models were trialled unmodified or after trimming to remove residues with a pLDDT confidence estimate of less than 70. In each case, the pLDDT values of the structure were converted to pseudo-*B* factors (Oeffner *et al.*, 2022 ▸). For some multi-domain targets where these trials failed, *Slice’N’Dice* (SnD; Simpkin *et al.*, 2022 ▸) was used to split the model into distinct structural units. A description of how this was performed is given in Section 2.5.2 below (MR and refinement). SnD allows selection from a number of algorithms for structural domain splitting: here, the Birch algorithm was used by default (balanced iterative reducing and clustering using hierarchies; Zhang *et al.*, 1996 ▸), with splitting based on the AF2 predicted aligned error (PAE; Jumper *et al.*, 2021 ▸) also being tested in some cases. The Birch algorithm amounts to applying clustering on the C^α^ coordinates of the input structure, whereas the PAE-based method generates clusters from the PAE matrix output from AF2, *AlphaFold*3 and *ESMFold*. The PAE matrix displays the predicted alignment error to the true structure for each residue pair. Contiguous regions of this matrix having a low PAE usually represent a domain or structural unit. Where single-molecule AF2 and CF predictions failed to yield a solution, *ESMFold* models (Lin *et al.*, 2023 ▸) and multimeric search models from *AlphaFold-Multimer* (Evans *et al.*, 2021 ▸) or *Uni-Fold Symmetry* (Li *et al.*, 2022 ▸) were subjected to the same pLDDT-based processing.

### 2.4. Secondary structure-based search models

*ARCIMBOLDO_LITE* (Rodríguez *et al.*, 2009 ▸) was used to try to solve selected α-helix-rich targets. *AMPLE* helical ensemble search models (Sánchez Rodríguez *et al.*, 2020 ▸) were also trialled in some cases. *ARCIMBOLDOcoiled_coil* mode was also selected for those targets predicted to contain coiled-coil regions.

### 2.5. Molecular replacement and refinement

#### 2.5.1. First pass: automated tests

Predictions were created using local installations of both AF2 (version 2.3.2) and CF (version 1.5.1) and passed into an automated structure-solution pipeline. This pipeline was based on applications from the *CCP*4 suite (version 8.0.016; Agirre *et al.*, 2023 ▸). Models were prepared as search models for MR using SnD to truncate residues with a pLDDT of <70 but not performing any splitting. MR was carried out using *Phaser* (McCoy *et al.*, 2007 ▸; Read & McCoy, 2016 ▸). The resulting solution was subjected to 100 cycles of jelly-body refinement in *REFMACAT* (Yamashita *et al.*, 2023 ▸). *Phaser* was run with default options. The assumed similarity of the search models to the target was set to an r.m.s.d. of 1.2 Å. Assessment of the solution was performed using a map correlation coefficient (map CC) calculated between the placed model(s) and a map calculated from the deposited structure. This was performed using *phenix.get_cc_mtz_pdb* from the *Phenix* suite (Liebschner *et al.*, 2019 ▸). A global map CC of greater than 0.25 was considered to be indicative of correct placement of the search model(s). Where a manually driven structure solution was required (described below), the *Phaser* LLG (log-likelihood gain) and TFZ (translation-function *Z*-score) were also used to assess the MR solutions. An LLG of better than 64 and a TFZ of better than 8.0 for each search model in MR are considered to be indicative of correct placement (Oeffner *et al.*, 2018 ▸).

#### 2.5.2. Second pass: manual structure solution

Where the automated tests failed to give a solution meeting the map CC threshold for a solution, test-case data and predictions were imported into a *CCP*4 Cloud project (Krissinel *et al.*, 2022 ▸) where search-model preparation, MR and subsequent steps could be carried out interactively through the interface. If multimer and *ESMFold* predictions were tested in these cases, the predictions were generated online using the DeepMind AF2 Google Colab server, the *Uni-Fold* Google Colab server and the *ESMFold* prediction service provided through the ESMAtlas website. These were then imported and prepared for MR in the *CCP*4 Cloud project. Solution attempts using *ARCIMBOLDO* were also run through the *CCP*4 Cloud interface. *AMPLE* tests were run from the command line. Where model splitting was required, several splits were tested. This typically involved splitting the initial prediction into two, three or more parts with SnD and passing all parts as separate search models to a *Phaser* MR run. The tree-like project-structure layout of *CCP*4 Cloud made it easy to explore each of the splitting strategies. In some cases, the parameterization of *Phaser* was adjusted to aid the determination of a correct solution. For example, sometimes the assumed r.m.s.d. of the search model to the target needed to be decreased from the chosen default of 1.2 Å to enable the correct placement of search models that were more accurate but constituted only a small part of the overall scattering content in the crystal. In other cases, only partial solutions could be found in MR, so additional refinement in *REFMACAT* and model building using *ModelCraft* (Bond & Cowtan, 2022 ▸) were run to verify the solution and bring it to a more complete state.

## 3. Results and discussion

### 3.1. Most recent SAD-phased structures can be solved with unedited or pLDDT-edited AF2 or CF models

**Figure 1:**
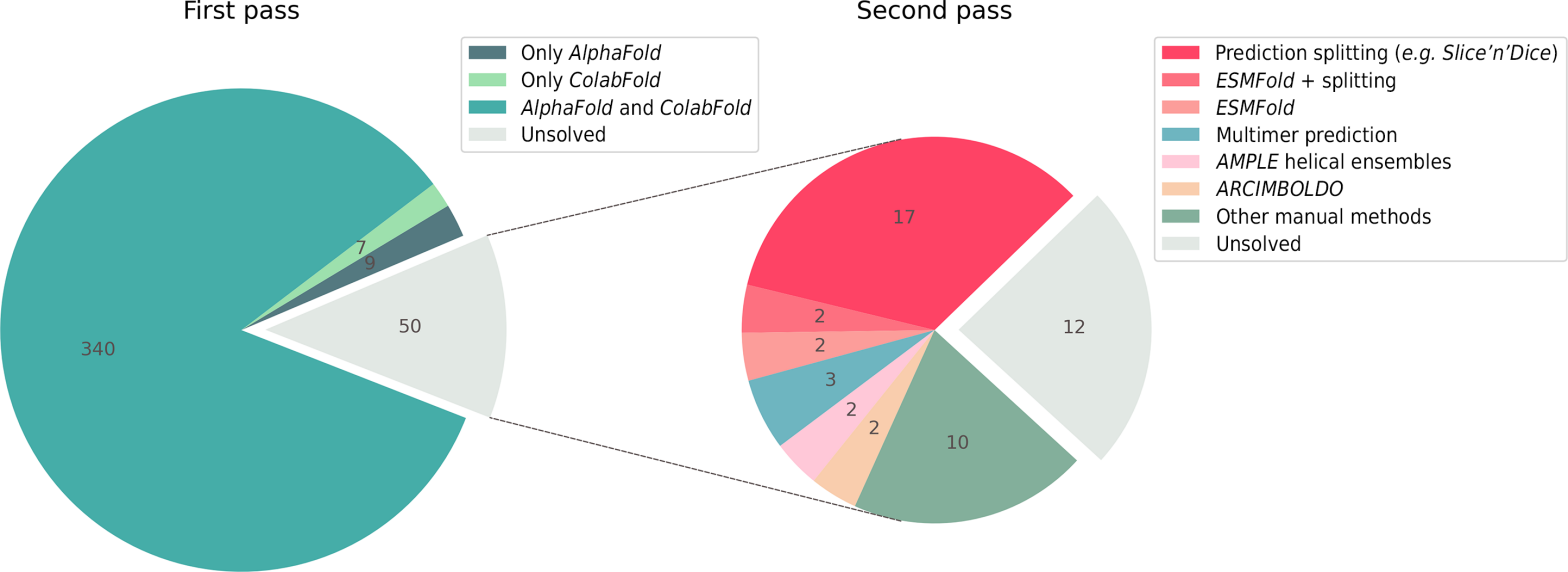
Pie charts showing the results from testing our SAD data set (406 structures). The pie chart on the left shows the results from the first pass where models from *ColabFold* and *AlphaFold* were run in MR with minimal modification (pLDDT ≥ 70 and conversion of pLDDT to predicted *B* factor); 356 of the 406 structures (87.7%) were solved in this first pass. The pie chart on the right shows the results from the second pass where various MR strategies were used on 50 cases that we had been unable to solve in the first pass. A further 38 cases were solved, bringing the total to 394/406 (97%).

We first attempted structure solution using DeepMind AF2 models, either unmodified or after trimming to remove residues with a pLDDT confidence estimate of less than 70. In each case, the pLDDT values of the structure were converted to *B* factors as in Section 2. Using a map CC of at least 0.25 (see Section 2) as a threshold, a large majority of the test cases achieved this criterion (349/406 = 86%), solving straightforwardly (Fig. 1 ▸). Recalling that these are not PDB depositions as a whole, but rather those for which SAD was used for phasing, this is a remarkable result that echoes previous work demonstrating the profound impact of AF2 on crystal structure solution by MR (Terwilliger *et al.*, 2023 ▸).

#### 3.1.1. *ColabFold* solves a slightly different set to DeepMind *AlphaFold*2

The development of the *ColabFold* implementation of AF2 (Mirdita *et al.*, 2022 ▸) has contributed significantly to the democratization of the technology. By replacing the original MSA-generation step of AF2 with a quicker approach based on *MMseqs*2 (Steinegger & Söding, 2017 ▸) and, in particular, by the provision of a fast API for the MSA-generation step, the CF developers have facilitated AF2 modelling on more modest hardware and, notably, on Colab pages. In most cases, AF2 and CF each produce excellent models which can be used largely interchangeably for downstream applications. However, we note here that the different MSAs generated by AF2 and CF can lead to subtly different structures with, in some cases, a crucial impact on MR success (Supplementary Table S1).

#### 3.1.2. Inclusion of templates is occasionally required for success

**Figure 2:**
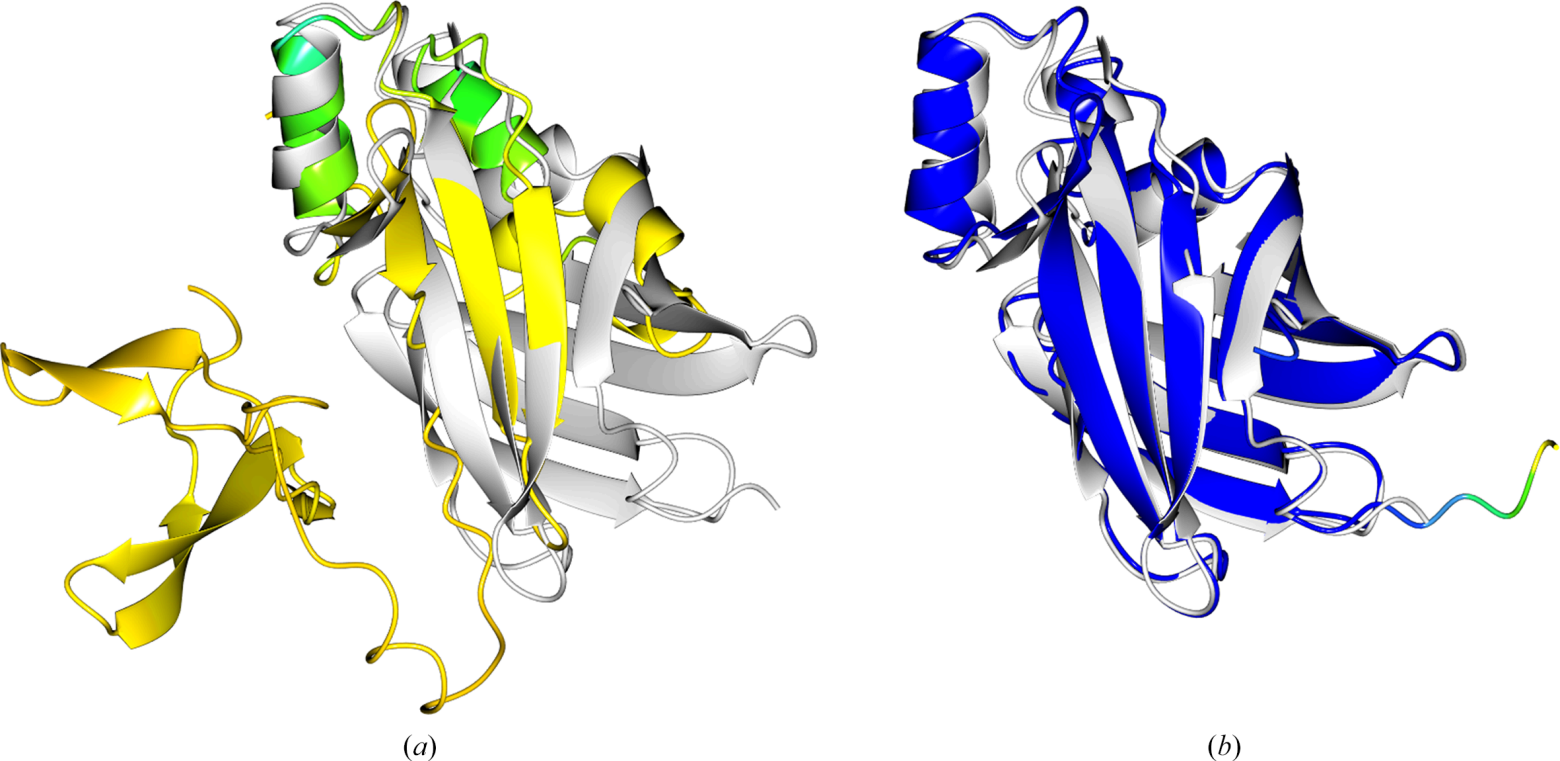
An alignment of the (*a*) *ColabFold* (r.m.s.d. 2.7 Å) and (*b*) AF2 (r.m.s.d. 1.3 Å) predictions coloured by pLDDT with the deposited structure PDB entry 7snr (2 Å resolution, two copies in the asymmetric unit, space group *P*2_1_2_1_2; white). The AF2 prediction benefitted from the use of a template during the prediction process to give a much higher confidence, and subsequently more accurate, prediction that was suitable as a search model in MR. All structural figures were created using the *CCP*4*mg* application (McNicholas *et al.*, 2011 ▸).

The AF2 pipeline employed here used templates where available. Although not required in most cases, in which the available MSA depth and the power of the modelling network combine to produce excellent models, AF2 and CF can optionally use information from homologous templates. A dramatic difference in the quality of the AF2 and CF models for PDB entry 7snr was noted (Fig. 2 ▸). It could be explained by the exceedingly shallow MSA produced by the CF pipeline (Neff/length = 0.01), which allows the extraction of only weak evolutionary covariance information. The AF2 model was far superior since, unlike the CF prediction, it used information from the PDB structures (PDB entries 5b0u, 4azn, 5ur9 and 4lkp) as templates.

### 3.2. Most of the remaining 50 structures yield to structure solution using computational methods

The 50 structures that were not straightforwardly solved were examined in more detail (Supplementary Tables S2–S7). As described in Section 2.5.2, alternative computational approaches were trialled, principally the splitting of AF2 models more finely into larger numbers of structural units. On a case-by-case basis, other approaches included *AlphaFold*2-*Multimer*, *Uni-Fold* (especially the symmetry-aware version) and *ESMFold*. MR with ideal helices in *ARCIMBOLDO_LITE* (Rodríguez *et al.*, 2009 ▸) or helical ensembles in *AMPLE* (Sánchez Rodríguez *et al.*, 2020 ▸) was also attempted in selected cases. Supplementary Tables S2–S7 summarize the key characteristics of these cases and indicate whether and how successful MR was achieved. Below, we consider some of the main findings and provide illustrative examples in each case.

#### 3.2.1. Automated splitting into two or more units solves many more cases

It is well understood that multi-domain proteins represent a challenge, even for the latest generation of modelling software. Inter-domain information from evolutionary covariance will likely be weaker than intra-domain information and, of course, flexible linkers between domains can lead to proteins sampling conformations with a variety of inter-domain orientations. In such cases the prediction may be valid as biologically accessible but only a representative conformation that may differ significantly from the crystallized conformation. Recognizing this issue, it is relatively commonplace to split domains of larger proteins, and the PAE, as provided by many methods, provides a readout of confidence in inter-domain orientations that can be used for splitting. Targets that yielded to this approach are listed in Supplementary Table S2. Here, we first applied the default PAE-independent Birch algorithm for domain splitting by SnD: this method allows the processing of experimental structures and structure predictions from any method.

**Figure 3:**
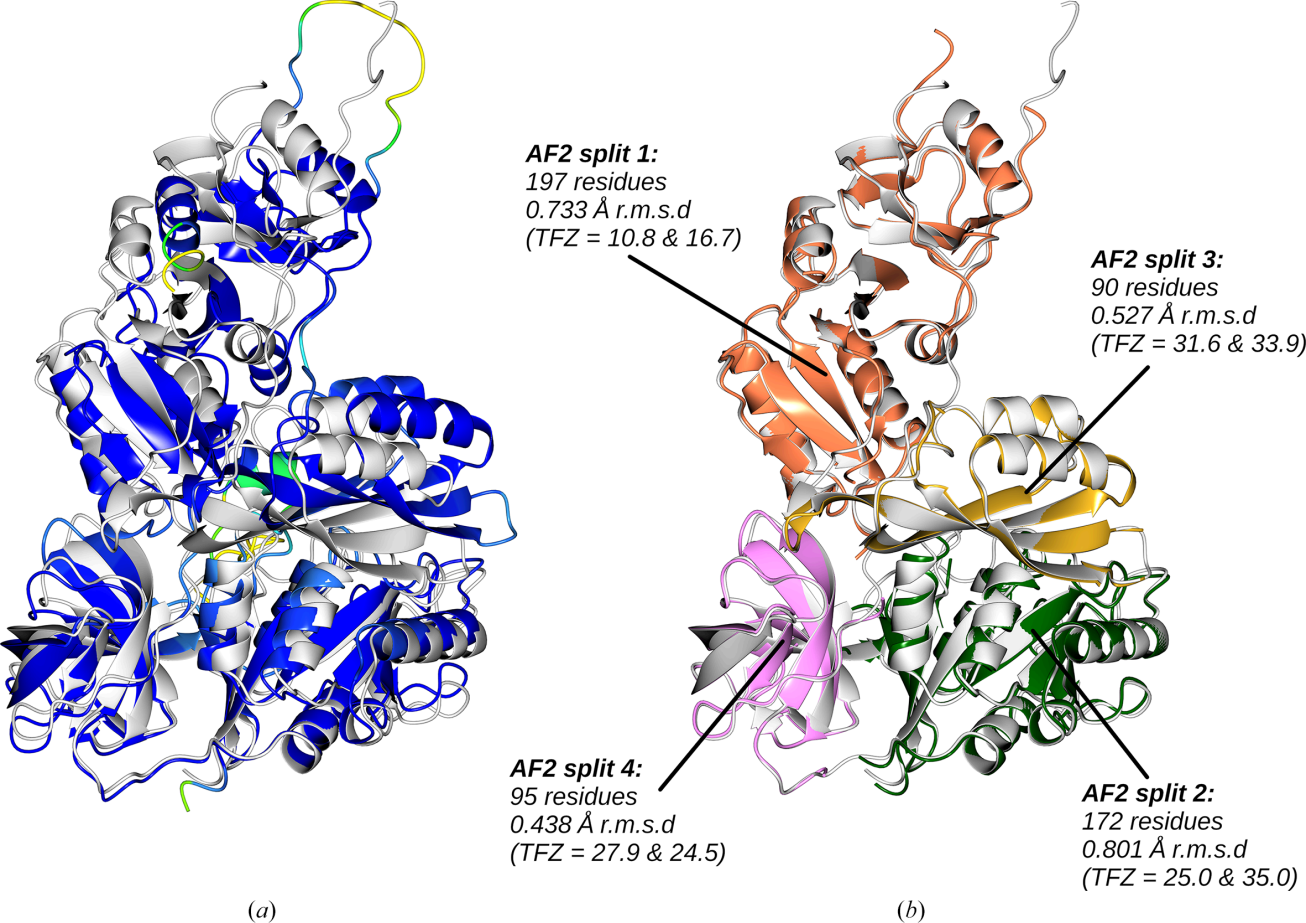
PDB entry 8ewh (2.33 Å resolution, two copies in the asymmetric unit, space group *P*2_1_). (*a*) shows the unmodified AF2 prediction (coloured by pLDDT) aligned with the crystal structure (white) using *GESAMT* to perform the alignment (r.m.s.d. 2.5 Å). MR using the full predicted model was not successful. (*b*) shows the alignment with the true structure of a four-way split of the AF2 prediction after it has been truncated to residues with pLDDT ≥ 70. The Birch clustering method employed in SnD was used to cluster the C^α^ atoms into the four clusters. *Phaser* successfully placed all eight fragments making up the two copies of the molecule in the asymmetric unit with a final LLG of 3458. TFZ values for each placed fragment are shown. To illustrate their accuracy, the r.m.s.d. for each split fragment to the crystal structure is also shown. The solution was then refined using 100 cycles of jelly-body refinement in *REFMACAT*, giving an *R* and *R*_free_ of 0.29 and 0.33, respectively. The solution was further improved to an *R* and *R*_free_ of 0.24 and 0.30, respectively, by automated model building using *ModelCraft*. This gave a global map CC of 0.772 when compared with the map generated from the deposited data.

An example of successful structure solution after Birch domain splitting is shown in Fig. 3 ▸ for the test case PDB entry 8ewh. After eliminating low-confidence residues (see Section 2), the structure prediction is split into four units which superimpose on the respective domains of the native structure much better than the original prediction. An MR search for the two copies of the overall molecule, *i.e.* the attempted placement of two copies of each of the four search models created by the splitting, resulted in the correct placement for all parts with an LLG of 3458. Subsequent jelly-body refinement with *REFMACAT* improved the solution to an *R* and *R*_free_ of 0.29 and 0.33, respectively.

#### 3.2.2. The splitting algorithm can be important

**Figure 4:**
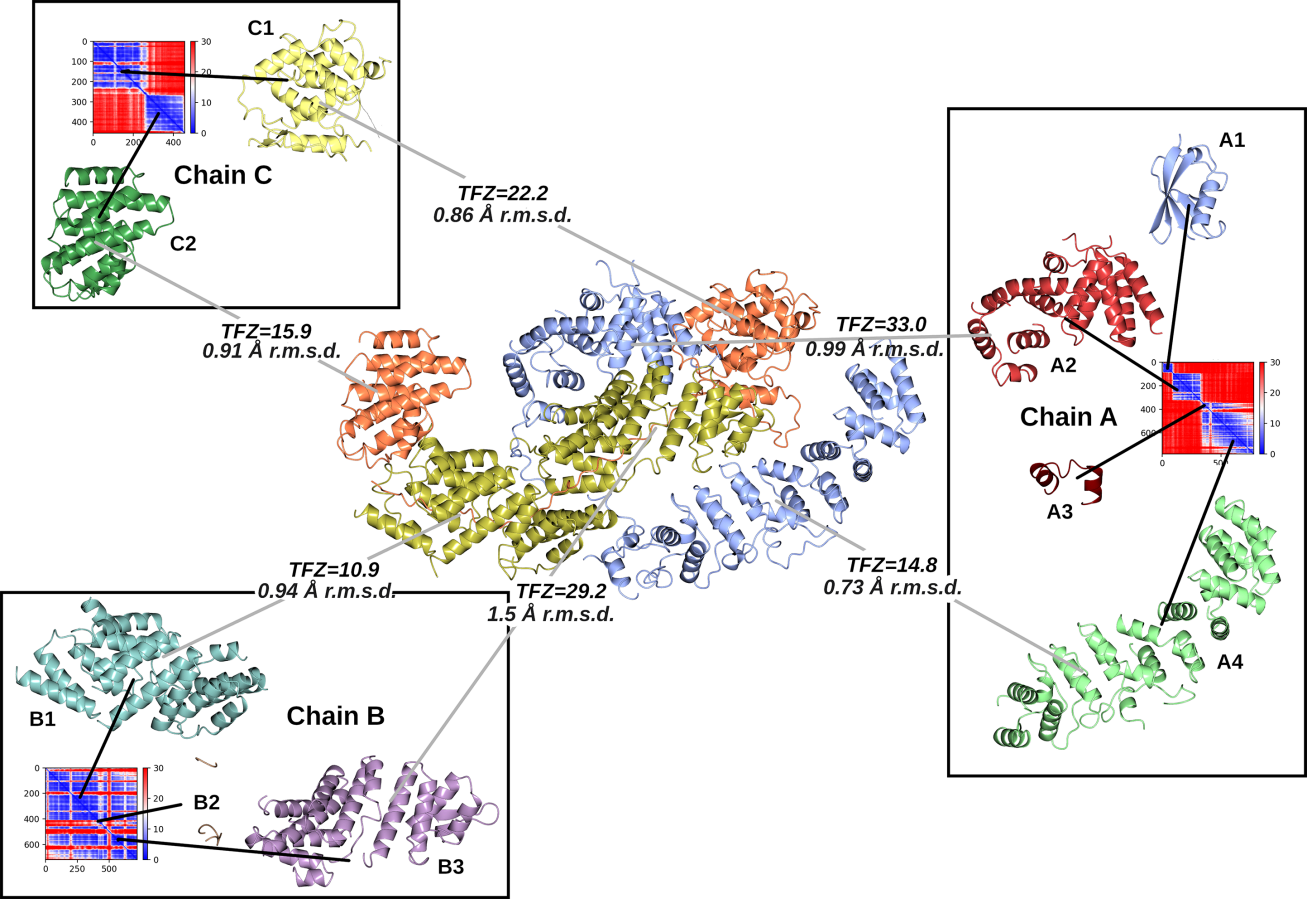
Solution of target structure PDB entry 8bfi (3 Å resolution, one copy in the asymmetric unit, space group *P*2_1_) using PAE-based domain splitting of *ColabFold* predictions of each of the three chains to create suitable search models for MR. The target structure is shown in the centre, coloured by its three chains: *A* (blue), *B* (gold) and *C* (orange). Chains *A* and *C* have domain-swapped parts, leading to large differences in the relative orientation of each of the domains of the predicted chain when compared with the crystallized form. However, utilizing the PAE matrices produced by *ColabFold* (shown for each chain; blue is low error and red is high error), the domains can be separated out from the chain predictions to produce accurate search models (shown in the boxes) that can be placed correctly in MR by *Phaser*. All predicted domains were placed correctly apart from *A*1, which was not present in the crystal, and *A*3 and *B*2, which both constituted a very small fraction of the overall scattering content. The TFZ from *Phaser* for each search-model placement is shown (values above 8 are indicative of correct placement). The final LLG for all of the placed components was 2048.

Domain partition of protein structures is an active area of research and a variety of approaches and programs are available (see, for example, Wells *et al.*, 2024 ▸; Kandathil *et al.*, 2024 ▸; Cretin *et al.*, 2022 ▸). The default Birch algorithm in SnD has the advantages of being quick and PAE-independent, meaning that it can be applied to any input structure. In some cases the Birch algorithm gives the best results (Supplementary Fig. S3): however, in other cases the use of alternative algorithms is required for success. For example, the target PDB entry 8bfi was not solved by SnD using Birch-defined partitions, but was solved using PAE-based domain splitting (Fig. 4 ▸)

#### 3.2.3. Alternative structure-prediction software can succeed

Recent CASP data have confirmed that AF2 predictions continue to underpin all of the most successful structure-prediction protocols. However, there is also strong interest in the alternative approach of structure prediction using protein language models (pLMs), especially for its much superior predictive speed, and CASP15 indeed showed that in a handful of cases the best-performing pLM method at the competition produced the most accurate model. Targets that yielded to this approach are listed in Supplementary Table S3.

**Figure 5:**
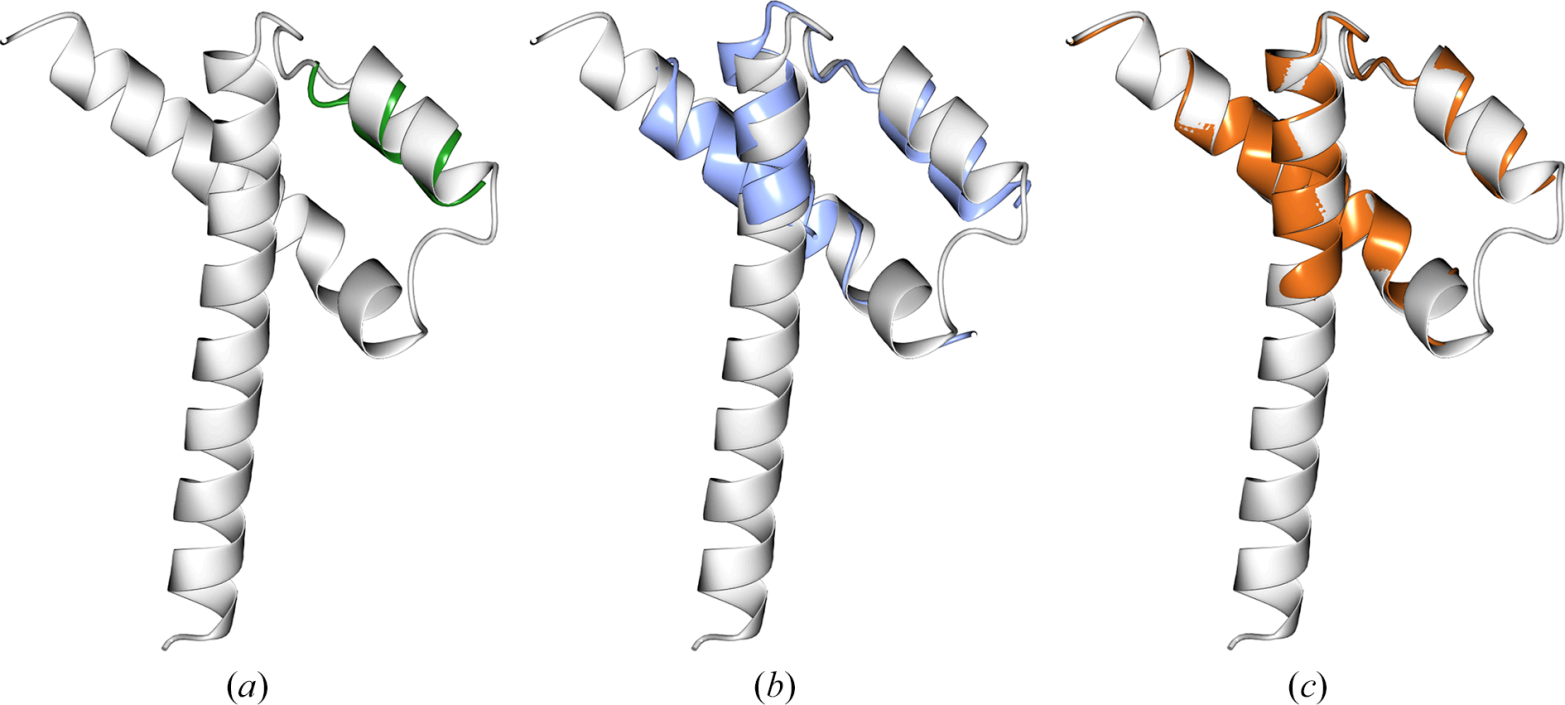
PDB entry 8bjw (2.39 Å resolution, six copies in the asymmetric unit, space group *P*3_2_21). Following model truncation to residues with pLDDT ≥ 70, the target structure (white) was aligned with (*a*) the AF2 prediction (green, 16 residues, r.m.s.d. 1.07 Å), (*b*) the *ColabFold* prediction (blue, 45 residues, r.m.s.d. 1.215 Å) and (*c*) the *ESMFold* prediction (orange, 60 residues, r.m.s.d. 0.503 Å).

For the case of PDB entry 8bjw, neither the AF2 or CF models succeeded with the approaches we tried, and nor did the target yield to MR with ideal helices in *ARCIMBOLDO_LITE*. However, a prediction from *ESMFold* processed to remove low-confidence residues did succeed (Fig. 5 ▸). In this case, removal of pLDDT < 70 residues from the AF2 model left only 16 residues which, given the six copies in the asymmetric unit and the moderate resolution, failed in MR. Interestingly, the processed *ESMFold* result (60 residues, 0.503 Å r.m.s.d.) is only a little larger and more accurate than the CF-derived search model (45 residues, 1.215 Å r.m.s.d.), but this marginal difference is evidently crucial. The final LLG for the six placed copies of the *ESMFold*-based search model was 655, with TFZ values for the placed copies ranging from 8.6 to 16.7. With refinement and model building, *R* and *R*_free_ were 0.22 and 0.32, respectively.

**Figure 6:**
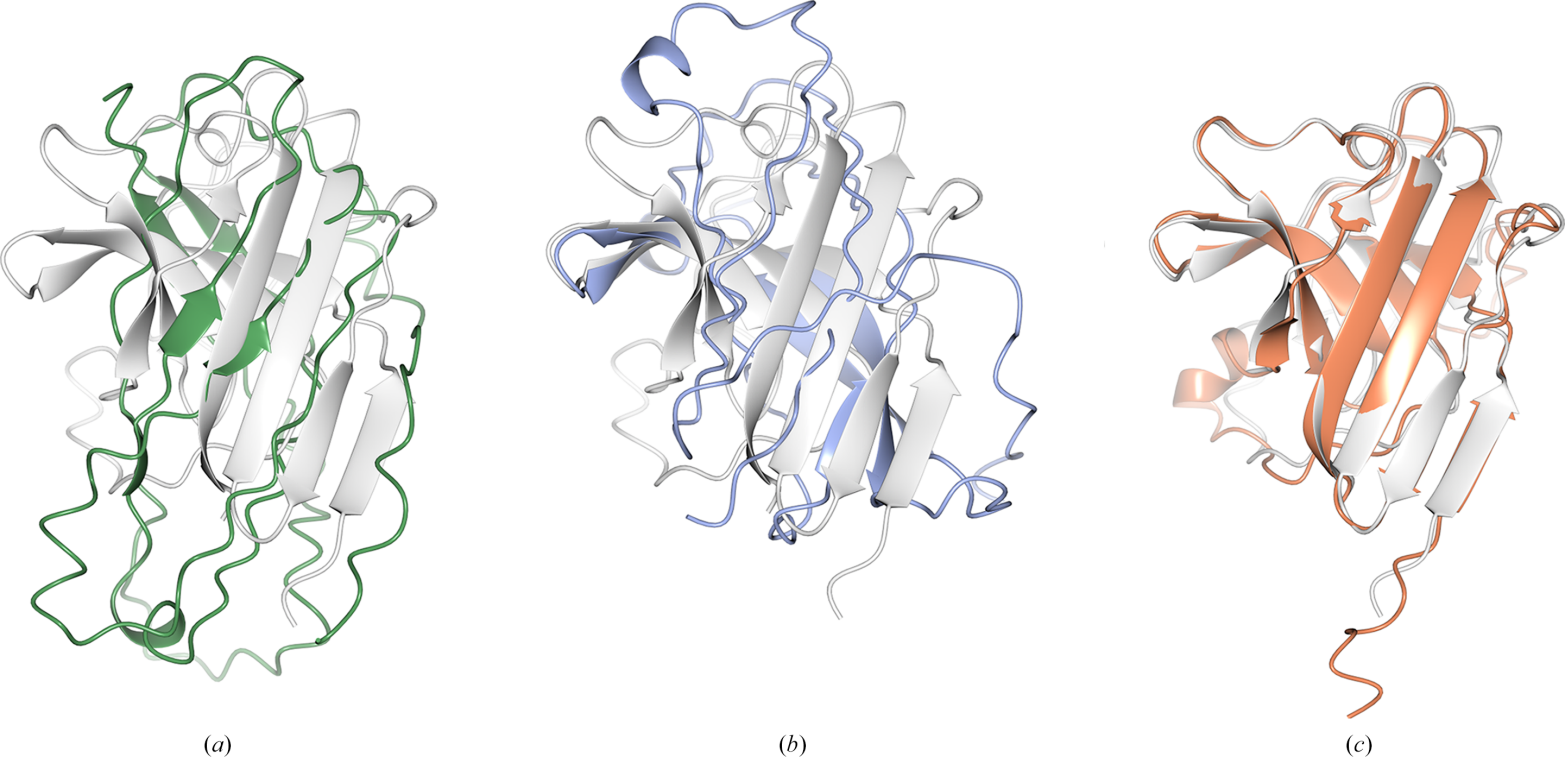
PDB entry 7qrr (1.9 Å resolution, 12 copies in the asymmetric unit, space group *C*121). The target structure (white) was aligned with (*a*) the AF2 prediction (green, r.m.s.d. 4.123 Å), (*b*) the *ColabFold* prediction (blue, r.m.s.d. 3.636 Å) and (*c*) the *ESMFold* prediction (orange, r.m.s.d. 1.384 Å).

The case of PDB entry 7qrr is even more remarkable. The AF2 and CF models are each very poor (Fig. 6 ▸), even when modelling the native trimer (data not shown), presumably due to the low Neff/length values of their input MSAs (0.01 in each case). The *ESMFold* model, however, is excellent and a remarkable 12 copies can be placed to solve the structure (LLG = 2064). Interestingly, PDB entries 7qrr and 8bjw are both virus proteins, the rapid sequence divergence of which during evolution is known to make the collection of homologous sequences more difficult (Karlin, 2024 ▸). Conceivably, in these cases the *ESMFold* language-model training more successfully inferred relationships across these divergent families than were discovered during the MSA generation of AF2 and CF modelling.

In the remaining two cases, PDB entries 8bvl and 8gxl, splitting of *ESMFold* predictions with SnD was required for structure solution.

#### 3.2.4. Modelling an oligomer can be helpful

In some cases it can be critical that the search model contains a significant proportion of the scattering matter, for example where data only extend to a relatively low resolution. Thus, the construction of oligomeric search models can help in these scenarios, giving the search model a larger signal in the MR search and enabling the correct placement to be found more readily. The latest generation of structure-prediction software can straightforwardly model protein complexes (Evans *et al.*, 2021 ▸) and, in some cases (Li *et al.*, 2022 ▸), can exploit noncrystallographic symmetry that might be apparent, for example from a self-rotation function.

**Figure 7:**
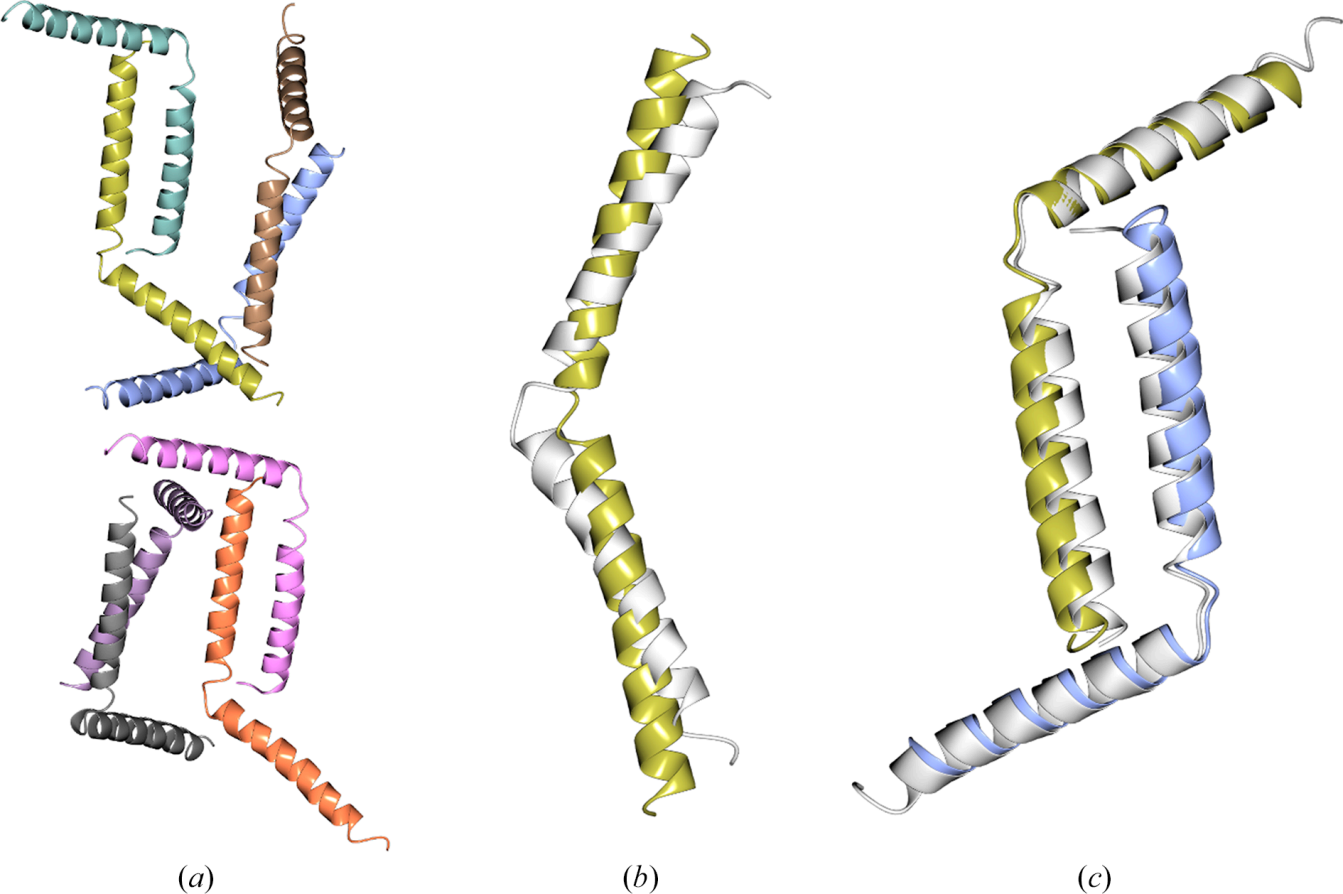
PDB entry 8h8h (2.7 Å resolution, eight copies in the asymmetric unit, space group *P*2_1_2_1_2_1_). (*a*) shows the entire content of the asymmetric unit, (*b*) shows a structural alignment of the AF2 prediction and a single chain from the deposited structure (r.m.s.d. 3.27 Å) and (*c*) shows a structural alignment of a dimer from the deposited structure (white) and a dimeric model generated using *Uni-Fold Symmetry* (r.m.s.d. 1.49 Å). Both the AF2 prediction and the *Uni-Fold Symmetry* dimer have been truncated to residues with pLDDT ≥ 70. The AF2 prediction failed to yield a solution in MR, either as the whole monomer or as a set of search models derived through splitting at the hinge between the helices. Both the dimer and the monomer produced as part of the *Uni-Fold Symmetry* dimer prediction can be used to determine the structure.

In the set used here, there were three cases (two from *Uni-Fold Symmetry* and one from *AlphaFold-Multimer*) in which the construction of an oligomer was absolutely essential (Supplementary Table S4). For example, Fig. 7 ▸ shows the case of PDB entry 8h8h, where a monomeric structure was insufficiently accurately captured. In this case modelling the *Uni-Fold Symmetry* dimer produced a more accurate model, with the angles between the two helices closer to those in the native structure. The resulting dimer can be used to solve the structure, but required some manual assistance to find all four copies. Three copies were initially placed by *Phaser*, achieving an LLG of 220. Refinement of this solution and the subsequent use of one of the refined dimers as a search model enabled the fourth copy to be placed correctly (TFZ = 34.9). The better formed monomer produced in the dimer prediction gives a solution more easily, with all eight copies found by *Phaser*, achieving an LLG of 1427.

**Figure 8:**
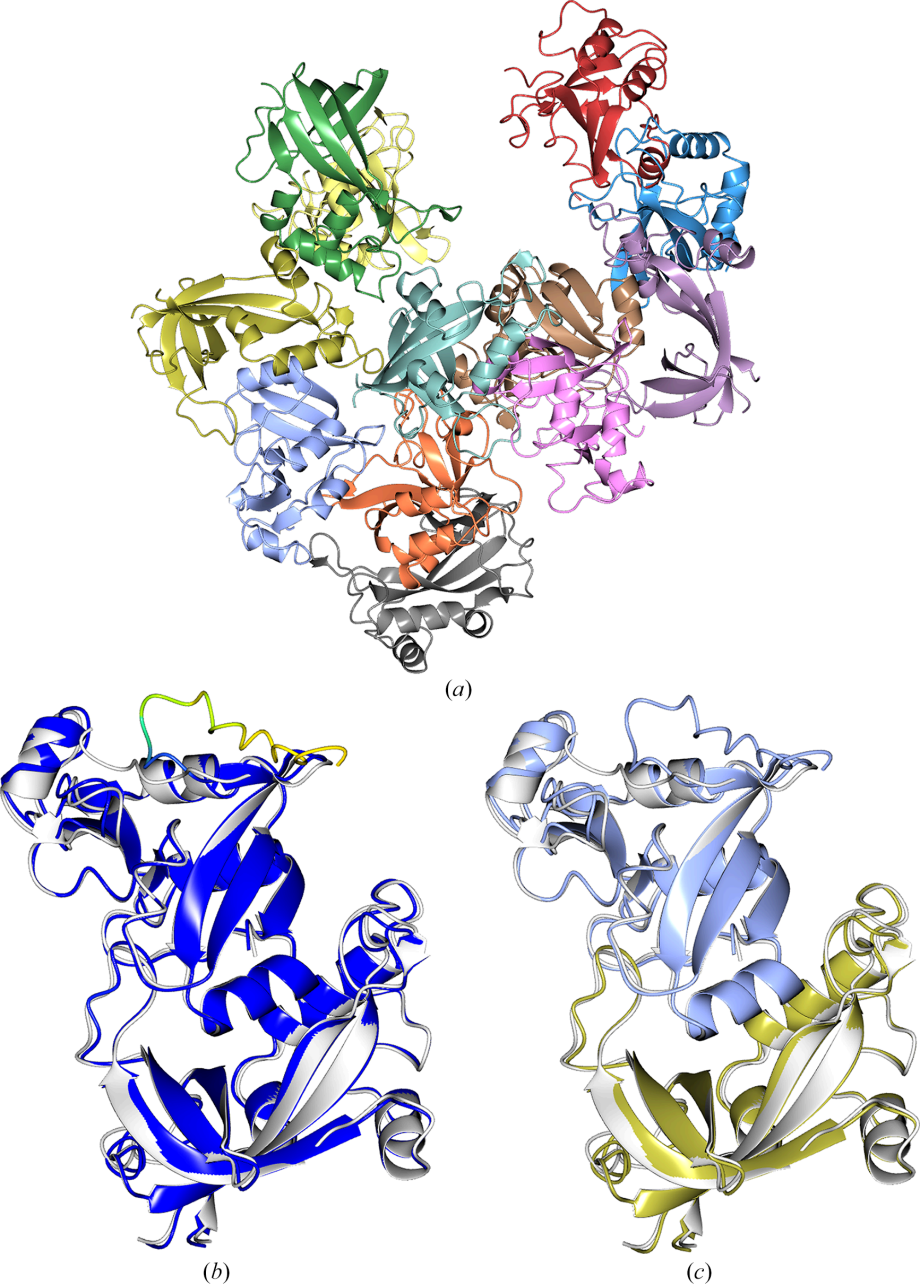
PDB entry 7rt7 [2.29 Å resolution, 12 copies in the asymmetric unit (6 + 6), space group *P*3_2_21]. The asymmetric unit consists of six copies of a heterogeneous complex of two chains (*a*). The AF2 prediction for the complex coloured by pLDDT aligned with the true structure of the complex (white) is shown in (*b*), while (*c*) displays the same alignment with the predicted complex coloured by chain. The r.m.s.d. for the complex aligned with the target is 0.735 Å. A solution could be found with *Phaser* using the individual chains as search models (taking 2 h on four CPU cores). However, using the complex search model gave a clearer signal for the correct positioning of the models, enabling a solution to be found in less than 15 min.

The case of PDB entry 7rt7, where the crystal contains six copies of a two-chain heterogeneous complex, demonstrates the advantage of using an *AlphaFold-Multimer* complex prediction rather than the individual monomers in MR. Although the monomeric search models can be used in MR successfully, the stronger signal for the complex search model containing the two chains solves the structure much more quickly (15 min versus 2 h; Fig. 8 ▸).

#### 3.2.5. Secondary-structure-based search models are still useful for coiled-coil and other helix-rich cases

As was evident at CASP15 (Simpkin, Mesdaghi *et al.*, 2023 ▸), even the latest modelling software can struggle with certain cases, most notably those for which few homologues are available by database search (and for which, of course, there is no template in the PDB). For these proteins, AF2 and similar tools have neither the template nor the evolutionary covariance information required to generate an initial model. Here, it must be recalled that AF2 is not the only tool in the box, and previous generations of unconventional MR software can still be deployed.

**Figure 9:**
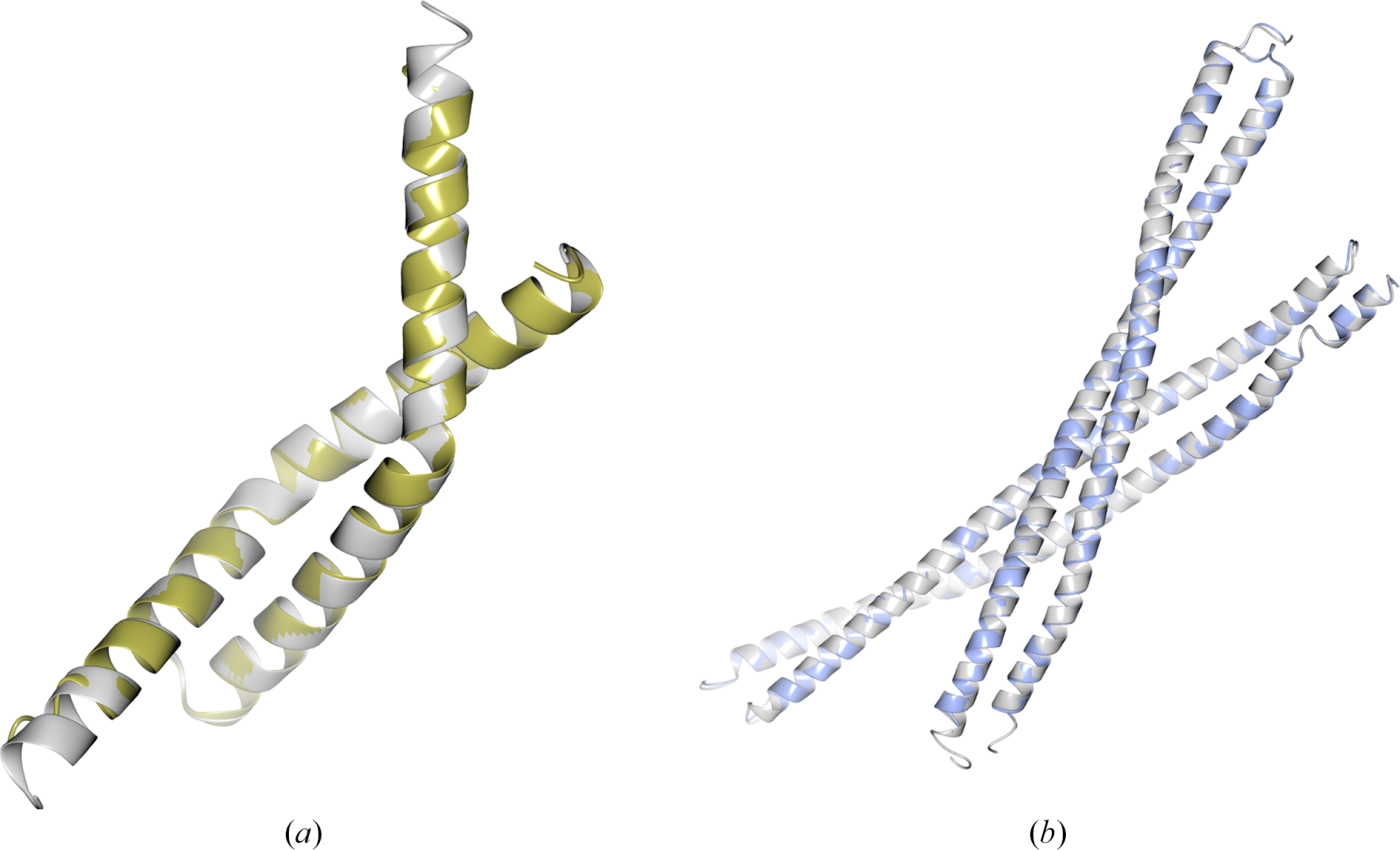
(*a*) The *ARCIMBOLDO* solution (gold) shown against the crystal structure (grey) for PDB entry 7twd (2.11 Å resolution, two copies in the asymmetric unit, space group *P*6_1_22). (*b*) The *AMPLE* solution (blue) shown against the crystal structure (grey) for PDB entry 8fby (2.4 Å resolution, four copies in the asymmetric unit, space group *P*2_1_2_1_2_1_).

We first used *ARCIMBOLDO_LITE* (Sammito *et al.*, 2013 ▸), which solved PDB entries 7twd and 7qwe (Supplementary Table S5). In the case of PDB entry 7twd, where each chain is a single, kinked α-helix, the helical twist is incorrectly modelled by AF2 and CF. PDB entry 7qwe is the structure of a *de novo* protein containing a homopentamer. AF2, CF and *Uni-Fold* models of the single chain or oligomers failed, but *ARCIMBOLDO_LITE* solved the structure (Fig. 9 ▸).

For a handful of cases of structures that were rich in α-helical content but that could not be solved any other way, one containing a coiled coil, we attempted MR with *AMPLE* using libraries of helical ensembles, which have been shown to outperform single ideal helical structures (Sánchez Rodríguez *et al.*, 2020 ▸). For two targets, PDB entries 8bc5 and 8fby, successful MR results were thereby obtained with search models of 30 and 25 residues, respectively (Supplementary Table S6). Interestingly, PDB entry 8bc5 featured as target T1122 at CASP15, where the extremely low solvent content of the crystal was noted as a feature that aggravated the high degree of difficulty imparted by the shallow MSA (Simpkin, Mesdaghi *et al.*, 2023 ▸).

Recognizing the presence of coiled-coil cases in the unsolved set, we tried the *ARCIMBOLDOcoiled_coil* protocol on seven cases, PDB entries 7sfy, 7uv8, 7w91, 8auc, 8b8d, 8jpa and 8tv0, but none were solved.

#### 3.2.6. Further investigation and intervention was required in some cases

**Figure 10:**
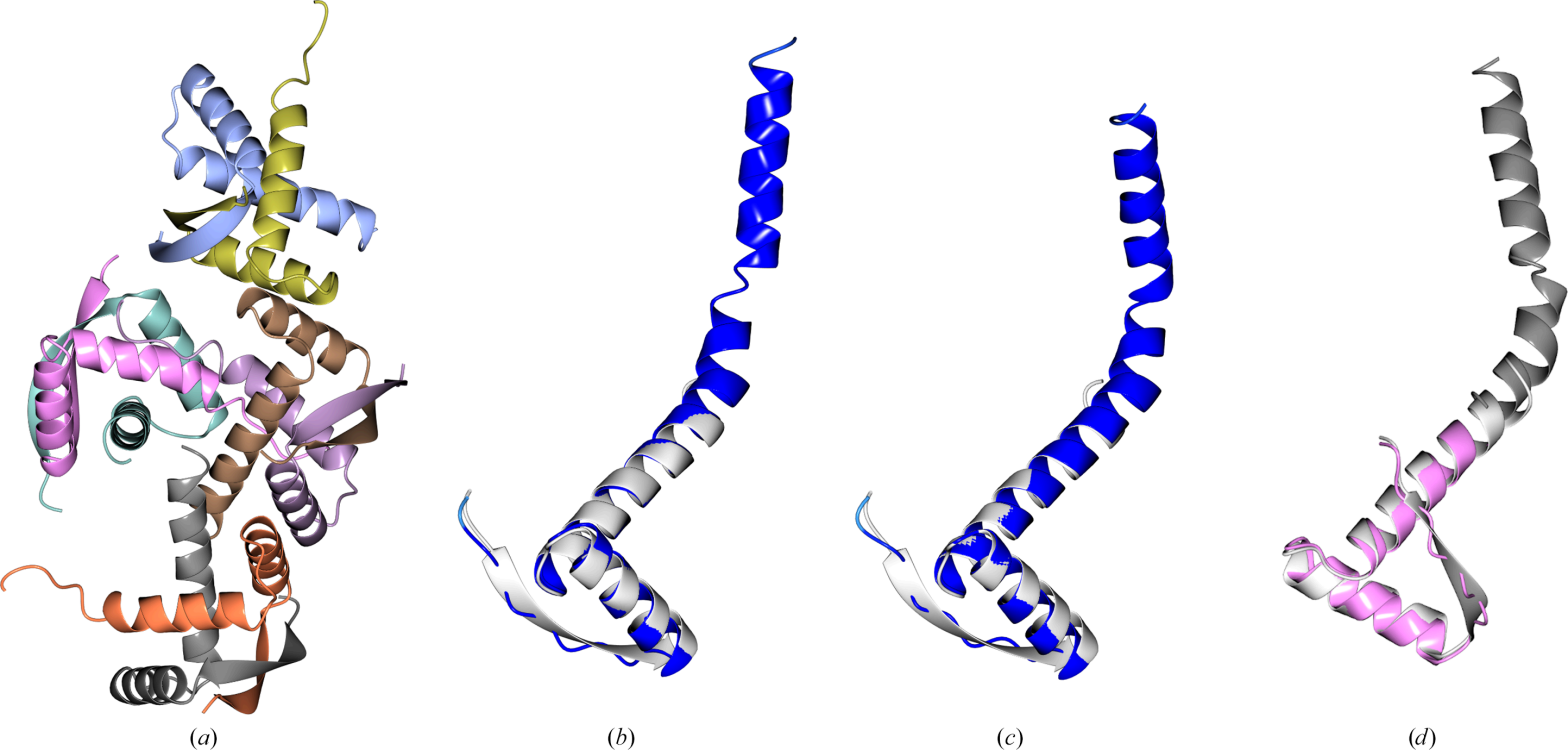
(*a*) PDB entry 8u12 (1.6 Å resolution, eight copies in the asymmetric unit, space group *H*3). (*b*) and (*c*) show the alignment of the predicted model from AF2 (r.m.s.d. 0.79 Å) and CF (r.m.s.d. 0.94 Å) onto a single chain from the target (white), respectively. The predicted models are coloured by pLDDT. Note that the predicted models contain about 25 additional C-terminal residues. These residues from the expected sequence (as deposited in the PDB) did not appear in the crystal. The structure was determined by splitting the model in two and finding eight copies of the N-terminal domain (*d*) (pink; r.m.s.d. 0.48 Å).

Where the straightforward manual processing approach in *CCP*4 Cloud failed to give a solution or gave only a partial solution, a more detailed examination of the case was sometimes required (Supplementary Table S7). One such case is PDB entry 8u12 (the crystal structure of antitoxin protein Rv0298), which consists of eight copies of the target protein in the asymmetric unit (Fig. 10 ▸*a*) in space group *H*3 with a resolution of 1.6 Å. 25 residues from the N-terminus of the expected sequence do not appear to have been crystallized, making MR using a predicted model based on this sequence difficult due to packing clashes between residues that are not present. In the initial MR trial using the entire predicted model from AF2 (Fig. 10 ▸*b*), *Phaser* struggled to find the correct placement for the models due to these packing clashes. This was followed by using SnD to split the model into two (Fig. 10 ▸*d*) and attempt to place eight copies of each part. Using these parts as search models, *Phaser* gave a more encouraging solution with an LLG of 2528. Closer examination of the result indicated that it had placed eight copies of one of the two split models (pink in Fig. 10 ▸*d*). Following MR with refinement in *REFMACAT* and model building using *ModelCraft*, the structure was close to completion (*R* = 0.25, *R*_free_ = 0.27), but it became clear that the 25 residues were missing from the N-terminus. This absence also meant that the solvent content was higher (67.6%) than that estimated using a Matthews coefficient calculation based on the initial sequence (52%).

#### 3.2.7. Some targets resist solution by any approach

**Figure 11:**
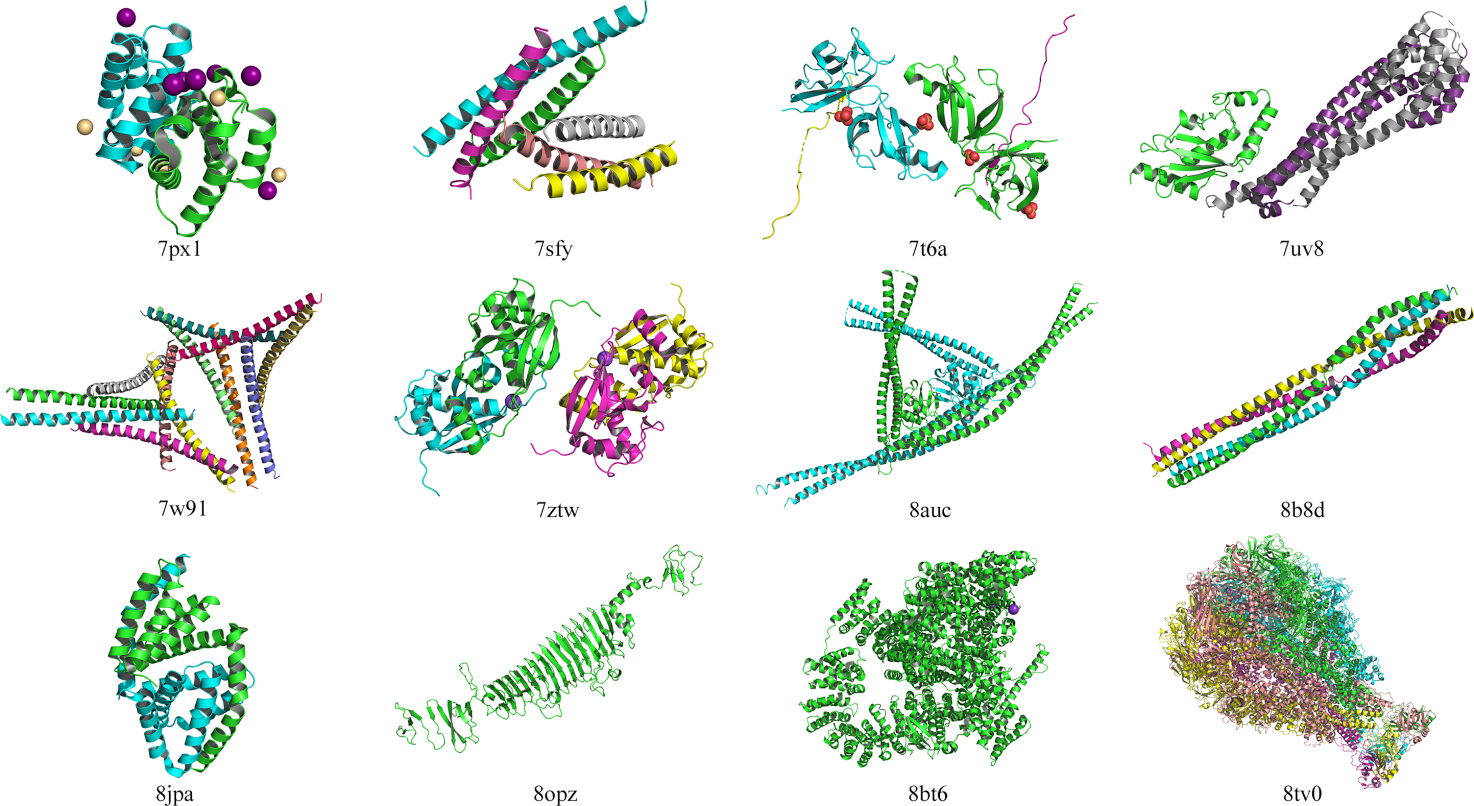
The crystal structures of the 12 cases that we were unable to solve with *ColabFold*/*AlphaFold*2 models or alternative MR methods. The structures are coloured by chain.

After application of the battery of tools described above, some targets were not solved using computational predictions (Table 1 ▸; Fig. 11 ▸). Of course, this does not preclude the possibility of some models being able to solve structures with extensive, expert intervention.

Inspection of Table 1 ▸ suggests that traditional complications of MR such as moderate diffraction resolution and the search model comprising only a small proportion of the scattering matter still apply in some cases. So, even a perfect model will fail in cases with many chains in the asymmetric unit (where a relevant biological oligomer cannot be accurately built) and insufficient resolution (Oeffner *et al.*, 2018 ▸).

Here, the focus is on model-centred factors that complicate MR. Given the overall excellent performance of AF2 and CF predictions as search models, but the understanding that modelling fails in cases with shallow MSAs, our initial prediction was that this set of recalcitrant targets would comprise largely orphan sequences or members of small families with few homologues in sequence databases. A rough minimum of 30 sequences in the MSA is often quoted as a threshold for successful modelling (Jumper *et al.*, 2021 ▸), but the diversity of the MSA is also important; hence the use of Neff/length here, where Neff is the number of effective (nonredundant to 80% sequence identity) sequences and the value is normalized by length. By this measure, 0.2 has been quoted as a threshold below which the MSA may be too shallow for good modelling (Ruiz-Serra *et al.*, 2021 ▸). The set of failing PDB entries in Table 1 ▸ covers 12 sequences. Of these, four lie below the Neff/length threshold in AF2 runs and eight in CF runs (Supplementary Table S9). For comparison, more than a third of the easily solved set had Neff/length values for CF runs of <0.2. This observation suggests that shallow MSAs are only part of the explanation of the difficulties encountered with this set.

Notably, seven of the 12 chains in Table 1 ▸ contain coiled-coil regions. This compares with only 42 of 355 (12%) in the set that were easily solved in an automated fashion (Supplementary Table S1). Furthermore, half of the structures that yielded only to ideal helices or helical ensembles (see above) contained coiled coils. These observations suggest that even with the current generation of modelling software, this architecture remains a continuing challenge in MR (Thomas *et al.*, 2015 ▸, 2020 ▸; Caballero *et al.*, 2018 ▸; Fu *et al.*, 2022 ▸).

Interestingly, among the most intractable of the recent CASP15 targets (Simpkin, Mesdaghi *et al.*, 2023 ▸), high α-helical content was also seen as aggravating: indeed, six of 14 chains (43%) in the 12 structures in Table 1 ▸ are defined as all-α. This compares with 42 all-α in the set of 356 (12%) that solved readily (Supplementary Table S1), suggesting that high helical content is indeed linked to AF2 target difficulty.

Finally, it is interesting to note that some targets (PDB entries 7px1, 7t6a and 7ztw) contain multiple metal ions: conceivably these, which are not considered explicitly by AF2, may have a significant influence on conformation as well as comprising, in some cases, a significant proportion of the scattering matter.

As this work was being finalized for publication, *AlphaFold*3 (AF3) was released (Abramson *et al.*, 2024 ▸) and structure solution of the 12 cases in Fig. 11 ▸ was attempted using AF3 models and the same methods. The only case that readily solved was PDB entry 8bt6 (Supplementary Fig. S2). AF3 produced a more accurate model that was therefore better split into structural units for MR. Note that in this single case an AF2 model was not available due to system limitations at the modelling stage.

## 4. Conclusions

It is already very apparent that the emergence of AF2 and similar tools has revolutionized the phasing step of X-ray crystallography. Already by far the most favoured approach, MR has been turbocharged by the ever-easier availability of high-quality structure predictions for most proteins. The literature contains many examples of structures that resisted structure determination until AF2 allowed easy phasing by MR (Barbarin-Bocahu & Graille, 2022 ▸). Larger-scale exercises, especially focused on CASP targets, have also shown that most crystal structures can be phased with AF2 models, even those ‘Free Modelling’ targets which have only weak sequence and structural similarities to PDB entries (Millán *et al.*, 2021 ▸; Pereira *et al.*, 2021 ▸; McCoy *et al.*, 2022 ▸; Simpkin, Mesdaghi *et al.*, 2023 ▸).

In this work, we focused on structures that were determined by SAD in the period July 2022 to October 2023. It is important to note that these are not representative of the PDB as a whole: in the same period, around 88% of structures were determined by MR. While there may be other reasons to choose SAD phasing, for example determination of bound ion positions, our focus is on a set of targets that is likely to include most targets that were not easily tractable by MR at the time. On the suggestion of a reviewer, we explored whether there was anything in the PDB that would have been able to solve the structures in our test set (Supplementary Table S8). We identified potential search models by using *MrParse* (Simpkin *et al.*, 2022 ▸) with *HHsearch* (Steinegger *et al.*, 2019 ▸) on the HHsearch database released prior to 27 July 2022, and by using *GESAMT* (Krissinel, 2012 ▸) to identify the most structurally similar model released prior to the deposition date for each target. MR using *Phaser* gave 99/406 solutions for the *MrParse* set and 87/406 solutions for the *GESAMT* set. When taken together, 145 of the 406 cases (35.6%) could have been solved using an existing structure in the PDB. Nonetheless, the remaining 261 cases were found to have met our criteria for targets that are not easily tractable by MR. By exploring these cases, we hoped to explore the outer boundaries of phasing with AF2 and other bioinformatics methods, and to reveal how many structures and what kind of structures require experimental phasing methods post-AF2. Notably, AF3, which became available at a very late stage, does not seem to change the picture dramatically with regard to providing search models for MR.

Our results show that a striking 356/406 or 87.7% of the set could be solved directly using an AF2 model, unprocessed except for the conversion of pLDDT confidence estimates to pseudo-*B* factors. In a further number of cases, elementary model editing to remove low-confidence regions, a procedure available in major software packages, enables structure solution. Also now routine, the splitting of a structure prediction into individual domains (Oeffner *et al.*, 2022 ▸; Simpkin *et al.*, 2022 ▸) was sometimes required for structure solution: however, in some cases success depended on the particular splitting algorithm used. In other cases, splitting domains was not essential but provided solutions that were significantly better quality, accelerating downstream rebuilding and refinement.

The use of methods other than AF2 was sometimes important. In four cases we found that neither flavour of AF2 succeeded, but an edited model from the protein language-model method *ESMFold* (Lin *et al.*, 2023 ▸) succeeded. In three other cases, the construction of a multimeric search model was required for success. As with search-model splitting, the use of a multimeric search model was sometimes advantageous for speed and for the better phases that resulted, even when a monomeric search model could solve a structure. Interestingly, oligomeric state can be predicted to some extent using deep-learning networks (Kshirsagar *et al.*, 2024 ▸). In four further cases tertiary-structure prediction failed to produce a successful search model, but MR with secondary-structure elements using *ARCIMBOLDO* (Rodríguez *et al.*, 2009 ▸) or *AMPLE* (Sánchez Rodríguez *et al.*, 2020 ▸) succeeded, emphasizing the continued utility of these methods in some cases.

Ultimately, these approaches collectively failed on 12 targets. One relevant feature is low MSA depth, *i.e.* some of these proteins have few homologues in the databases. This feature deprives AF2 of the evolutionary covariance information which (in the absence of templates, of course) is essential for the construction of an initial model (Jumper *et al.*, 2021 ▸). This mirrors other findings, such as those of recent CASPs (Pereira *et al.*, 2021 ▸; Simpkin, Mesdaghi *et al.*, 2023 ▸). This remains a current development frontier of predictive methods despite the possibilities of artificial MSA depth enhancement (Zhang *et al.*, 2023 ▸) and the means to recognize the fast evolution of viral proteins and detect more distant homologues than current pipelines do (Karlin, 2024 ▸). There are also recurrent suggestions that pLM-based methods may outperform MSA-based approaches in such cases (Chowdhury *et al.*, 2022 ▸; Wang *et al.*, 2022 ▸; Jing *et al.*, 2024 ▸), although this was not apparent at the CASP15 experiment (Simpkin, Mesdaghi *et al.*, 2023 ▸).

Also notable are the continued difficulties with coiled-coil proteins, whose unpredictable irregularities, oligomeric state and parallel versus antiparallel architectures remain, despite ongoing efforts (Madeo *et al.*, 2023 ▸; Madaj *et al.*, 2024 ▸), a significant hindrance to structure prediction and hence the use of modelled structures for MR.

Of course, a sufficiently accurate search model is necessary but not sufficient for structure solution by MR. Even with a good model available, a case may still fail where one or more traditional complications apply. These include cases where a large number of copies of the search model are present in the asymmetric unit, challenging crystal pathologies (twinning and tNCS) and crystals that diffract only to poorer resolution (≥3 Å). Thus, in terms of prioritizing cases for experimental phasing beamlines, difficult targets can initially be predicted bioinformatically, especially by assessing the depth and diversity of the MSA that can be constructed from homologous sequences, but other complications will only become evident once the crystal characteristics can be seen.

## Supplementary Material

List of PDB codes and Supplementary Figures.3. DOI: 10.1107/S2059798324009380/qu5006sup1.pdf

Supplementary Tables S1 to S9. DOI: 10.1107/S2059798324009380/qu5006sup2.xlsx

## Figures and Tables

**Table 1:** Information on the resolution, asymmetric unit content, size, secondary structure and crystal pathology of the PDB entries that we were unable to solve using *ColabFold*/*AlphaFold*2 or alternative MR methods This table is extended in Supplementary Table S9 to give details of the modelling.

PDB code	Resolution (Å)	No. of entities	No. of molecules in asymmetric unit per entity	Biological assembly	No. of residues in each entity	Secondary structure	*Socket*2: contains coiled coil?	Crystal pathology: contains twinning?	Crystal pathology: contains tNCS?
7px1	2.33	1	2	Homodimer	89	All helix	No	No	Yes
7sfy	2.5	2	4, 2	Heterotrimer	46, 42	All helix	Yes	No	No
7t6a	1.65	2	2, 2	Heterodimer	185, 44	Mostly sheet, all sheet	No, no	No	No
7uv8	2.7	2	1, 2	Heterotrimer	150, 212	Mostly helix, all helix	No, yes	No	No
7w91	3.29	1	12	Homohexamer	56	All helix	Yes	No	No
7ztw	1.93	1	4	Homodimer	150	Mostly helix	No	No	Yes
8auc	3.5	1	2	Monomer	600	Mostly helix	Yes	No	Yes
8b8d	2.4	1	4	Homotetramer	115	All helix	Yes	No	No
8jpa	2.3	1	2	Homodimer	183	All helix	No	No	Yes
8opz	1.5	1	1	Homotrimer	641	Mostly sheet	No	Yes	No
8bt6	2.33	1	1	Monomer	2646	Mostly helix	Yes	No	No
8tv0	3.1	1	5	Homopentamer	2537	Mixed	Yes	No	No

## References

[bb1] Abramson, J., Adler, J., Dunger, J., Evans, R., Green, T., Pritzel, A., Ronneberger, O., Willmore, L., Ballard, A. J., Bambrick, J., Bodenstein, S. W., Evans, D. A., Hung, C., O’Neill, M., Reiman, D., Tunyasuvunakool, K., Wu, Z., Žemgulytė, A., Arvaniti, E., Beattie, C., Bertolli, O., Bridgland, A., Cherepanov, A., Congreve, M., Cowen-Rivers, A. I., Cowie, A., Figurnov, M., Fuchs, F. B., Gladman, H., Jain, R., Khan, Y. A., Low, C. M. R., Perlin, K., Potapenko, A., Savy, P., Singh, S., Stecula, A., Thillaisundaram, A., Tong, C., Yakneen, S., Zhong, E. D., Zielinski, M., Žídek, A., Bapst, V., Kohli, P., Jaderberg, M., Hassabis, D. & Jumper, J. M. (2024). *Nature*, **630**, 493–500.

[bb2] Agirre, J., Atanasova, M., Bagdonas, H., Ballard, C. B., Baslé, A., Beilsten-Edmands, J., Borges, R. J., Brown, D. G., Burgos-Mármol, J. J., Berrisford, J. M., Bond, P. S., Caballero, I., Catapano, L., Chojnowski, G., Cook, A. G., Cowtan, K. D., Croll, T. I., Debreczeni, J. É., Devenish, N. E., Dodson, E. J., Drevon, T. R., Emsley, P., Evans, G., Evans, P. R., Fando, M., Foadi, J., Fuentes-Montero, L., Garman, E. F., Gerstel, M., Gildea, R. J., Hatti, K., Hekkelman, M. L., Heuser, P., Hoh, S. W., Hough, M. A., Jenkins, H. T., Jiménez, E., Joosten, R. P., Keegan, R. M., Keep, N., Krissinel, E. B., Kolenko, P., Kovalevskiy, O., Lamzin, V. S., Lawson, D. M., Lebedev, A. A., Leslie, A. G. W., Lohkamp, B., Long, F., Malý, M., McCoy, A. J., McNicholas, S. J., Medina, A., Millán, C., Murray, J. W., Murshudov, G. N., Nicholls, R. A., Noble, M. E. M., Oeffner, R., Pannu, N. S., Parkhurst, J. M., Pearce, N., Pereira, J., Perrakis, A., Powell, H. R., Read, R. J., Rigden, D. J., Rochira, W., Sammito, M., Sánchez Rodríguez, F., Sheldrick, G. M., Shelley, K. L., Simkovic, F., Simpkin, A. J., Skubak, P., Sobolev, E., Steiner, R. A., Stevenson, K., Tews, I., Thomas, J. M. H., Thorn, A., Valls, J. T., Uski, V., Usón, I., Vagin, A., Velankar, S., Vollmar, M., Walden, H., Waterman, D., Wilson, K. S., Winn, M. D., Winter, G., Wojdyr, M. & Yamashita, K. (2023). *Acta Cryst.* D**79**, 449–461.

[bb3] Barbarin-Bocahu, I. & Graille, M. (2022). *Acta Cryst.* D**78**, 517–531. 10.1107/S2059798322002157 35362474

[bb4] Berman, H., Henrick, K. & Nakamura, H. (2003). *Nat. Struct. Mol. Biol.* **10**, 980. 10.1038/nsb1203-980 14634627

[bb5] Bibby, J., Keegan, R. M., Mayans, O., Winn, M. D. & Rigden, D. J. (2012). *Acta Cryst.* D**68**, 1622–1631. 10.1107/S0907444912039194 23151627

[bb6] Bond, P. S. & Cowtan, K. D. (2022). *Acta Cryst.* D**78**, 1090–1098. 10.1107/S2059798322007732 PMC9435595 36048149

[bb7] Breugel, M. van, Rosa e Silva, I. & Andreeva, A. (2022). *Commun. Biol.* **5**, 312. 10.1038/s42003-022-03269-0 PMC8983713 35383272

[bb8] Caballero, I., Sammito, M., Millán, C., Lebedev, A., Soler, N. & Usón, I. (2018). *Acta Cryst.* D**74**, 194–204. 10.1107/S2059798317017582 PMC5947760 29533227

[bb9] Chowdhury, R., Bouatta, N., Biswas, S., Floristean, C., Kharkar, A., Roy, K., Rochereau, C., Ahdritz, G., Zhang, J., Church, G. M., Sorger, P. K. & AlQuraishi, M. (2022). *Nat. Biotechnol.* **40**, 1617–1623. 10.1038/s41587-022-01432-w PMC10440047 36192636

[bb10] Cretin, G., Galochkina, T., Vander Meersche, Y., de Brevern, A. G., Postic, G. & Gelly, J. C. (2022). *Nucleic Acids Res.* **50**, W732–W738. 10.1093/nar/gkac370 PMC9252838 35580056

[bb11] El Omari, K., Duman, R., Mykhaylyk, V., Orr, C. M., Latimer-Smith, M., Winter, G., Grama, V., Qu, F., Bountra, K., Kwong, H. S., Romano, M., Reis, R. I., Vogeley, L., Vecchia, L., Owen, C. D., Wittmann, S., Renner, M., Senda, M., Matsugaki, N., Kawano, Y., Bowden, T. A., Moraes, I., Grimes, J. M., Mancini, E. J., Walsh, M. A., Guzzo, C. R., Owens, R. J., Jones, E. Y., Brown, D. G., Stuart, D. I., Beis, K. & Wagner, A. (2023). *Commun. Chem.* **6**, 219. 10.1038/s42004-023-01014-0 PMC10570326 37828292

[bb12] Evans, R., O’Neill, M., Pritzel, P., Antropova, N., Senior, A., Green, T., Žídek, A., Bates, R., Blackwell, S., Yim, J., Ronneberger, O., Bodenstein, S., Zielinski, M., Bridgland, A., Potapenko, A., Cowie, A., Tunyasuvunakool, K., Jain, R., Clancy, E. Kohli, P., Jumper, J. & Hassabis, D. (2021). *bioRxiv*, 2021.10.04.463034.

[bb13] Fu, R., Su, W. & He, H. (2022). *Crystals*, **12**, 1674.

[bb14] Jing, X., Wu, F., Luo, X. & Xu, J. (2024). *Proc. Natl Acad. Sci. USA*, **121**, e2308788121. 10.1073/pnas.2308788121 PMC10990103 38507445

[bb15] Johnson, L. S., Eddy, S. R. & Portugaly, E. (2010). *BMC Bioinformatics*, **11**, 431. 10.1186/1471-2105-11-431 PMC2931519 20718988

[bb16] Jumper, J., Evans, R., Pritzel, A., Green, T., Figurnov, M., Ronneberger, O., Tunyasuvunakool, K., Bates, R., Žídek, A., Potapenko, A., Bridgland, A., Meyer, C., Kohl, S. A. A., Ballard, A. J., Cowie, A., Romera-Paredes, B., Nikolov, S., Jain, R., Adler, J., Back, T., Petersen, S., Reiman, D., Clancy, E., Zielinski, M., Steinegger, M., Pacholska, M., Berghammer, T., Bodenstein, S., Silver, D., Vinyals, O., Senior, A. W., Kavukcuoglu, K., Kohli, P. & Hassabis, D. (2021). *Nature*, **596**, 583–589. 10.1038/s41586-021-03819-2 PMC8371605 34265844

[bb17] Kandathil, S. M., Lau, A. M. & Jones, D. T. (2024). *bioRxiv*, 2024.03.25.586696.

[bb18] Karlin, D. G. (2024). *J. Gen. Virol.* **105**, https://doi.org/10.1099/jgv.0.001948. 10.1099/jgv.0.001948 38193819

[bb61] Krissinel, E. (2012). *J. Mol. Biochem.* **1**, 76–85. PMC5117261 27882309

[bb19] Krissinel, E., Lebedev, A. A., Uski, V., Ballard, C. B., Keegan, R. M., Kovalevskiy, O., Nicholls, R. A., Pannu, N. S., Skubák, P., Berrisford, J., Fando, M., Lohkamp, B., Wojdyr, M., Simpkin, A. J., Thomas, J. M. H., Oliver, C., Vonrhein, C., Chojnowski, G., Basle, A., Purkiss, A., Isupov, M. N., McNicholas, S., Lowe, E., Triviño, J., Cowtan, K., Agirre, J., Rigden, D. J., Uson, I., Lamzin, V., Tews, I., Bricogne, G., Leslie, A. G. W. & Brown, D. G. (2022). *Acta Cryst.* D**78**, 1079–1089. 10.1107/S2059798322007987 PMC9435598 36048148

[bb20] Kshirsagar, M., Meller, A., Humphreys, I., Sledzieski, S., Xu, Y., Dodhia, R., Horvitz, E., Berger, B., Bowman, G., Lavista Ferres, J., Baker, D. & Baek, M. (2024). *Research Square*, https://doi.org/10.21203/rs.3.rs-4215086/v1.

[bb21] Kumar, P. & Woolfson, D. N. (2021). *Bioinformatics*, **37**, 4575–4577. 10.1093/bioinformatics/btab631 PMC8652024 34498035

[bb22] Li, Z., Liu, X., Chen, W., Shen, F., Bi, H., Ke, G. & Zhang, L. (2022). *bioRxiv*, 2022.08.04.502811.

[bb23] Liebschner, D., Afonine, P. V., Baker, M. L., Bunkóczi, G., Chen, V. B., Croll, T. I., Hintze, B., Hung, L.-W., Jain, S., McCoy, A. J., Moriarty, N. W., Oeffner, R. D., Poon, B. K., Prisant, M. G., Read, R. J., Richardson, J. S., Richardson, D. C., Sammito, M. D., Sobolev, O. V., Stockwell, D. H., Terwilliger, T. C., Urzhumtsev, A. G., Videau, L. L., Williams, C. J. & Adams, P. D. (2019). *Acta Cryst.* D**75**, 861–877. 10.1107/S2059798319011471 PMC6778852 31588918

[bb24] Lin, Z., Akin, H., Rao, R., Hie, B., Zhu, Z., Lu, W., Smetanin, N., Verkuil, R., Kabeli, O., Shmueli, Y., dos Santos Costa, A., Fazel-Zarandi, M., Sercu, T., Candido, S. & Rives, A. (2023). *Science*, **379**, 1123–1130. 10.1126/science.ade2574 36927031

[bb25] Madaj, R., Martinez-Goikoetxea, M., Kaminski, K., Ludwiczak, J. & Dunin-Horkawicz, S. (2024). *bioRxiv*, 2024.03.07.583852.

[bb26] Madeo, G., Savojardo, C., Manfredi, M., Martelli, P. L. & Casadio, R. (2023). *Bioinformatics*, **39**, btad495. 10.1093/bioinformatics/btad495 PMC10425188 37540220

[bb27] McCoy, A. J., Sammito, M. D. & Read, R. J. (2022). *Acta Cryst.* D**78**, 1–13. 10.1107/S2059798321012122 PMC8725160 34981757

[bb28] McCoy, A. J., Grosse-Kunstleve, R. W., Adams, P. D., Winn, M. D., Storoni, L. C. & Read, R. J. (2007). *J. Appl. Cryst.* **40**, 658–674. 10.1107/S0021889807021206 PMC2483472 19461840

[bb29] McNicholas, S., Potterton, E., Wilson, K. S. & Noble, M. E. M. (2011). *Acta Cryst.* D**67**, 386–394. 10.1107/S0907444911007281 PMC3069754 21460457

[bb30] Millán, C., Keegan, R. M., Pereira, J., Sammito, M. D., Simpkin, A. J., McCoy, A. J., Lupas, A. N., Hartmann, M. D., Rigden, D. J. & Read, R. J. (2021). *Proteins*, **89**, 1752–1769. 10.1002/prot.26214 PMC8881082 34387010

[bb31] Mirdita, M., Schütze, K., Moriwaki, Y., Heo, L., Ovchinnikov, S. & Steinegger, M. (2022). *Nat. Methods*, **19**, 679–682. 10.1038/s41592-022-01488-1 PMC9184281 35637307

[bb101] Mirdita, M., von den Driesch, L., Galiez, C., Martin, M. J., Söding, J. & Steinegger, M. (2017). *Nucleic Acids Res.* **45**, D170–D176. 10.1093/nar/gkw1081 PMC5614098 27899574

[bb32] Oeffner, R. D., Afonine, P. V., Millán, C., Sammito, M., Usón, I., Read, R. J. & McCoy, A. J. (2018). *Acta Cryst.* D**74**, 245–255. 10.1107/S2059798318004357 PMC5892874 29652252

[bb33] Oeffner, R. D., Croll, T. I., Millán, C., Poon, B. K., Schlicksup, C. J., Read, R. J. & Terwilliger, T. C. (2022). *Acta Cryst.* D**78**, 1303–1314. 10.1107/S2059798322010026 PMC9629492 36322415

[bb34] Pereira, J., Simpkin, A. J., Hartmann, M. D., Rigden, D. J., Keegan, R. M. & Lupas, A. N. (2021). *Proteins*, **89**, 1687–1699. 10.1002/prot.26171 34218458

[bb35] Poon, B. K., Terwilliger, T. C. & Adams, P. D. (2024). *Protein Sci.* **33**, e4992. 10.1002/pro.4992 PMC11034488 38647406

[bb36] Qian, B., Raman, S., Das, R., Bradley, P., McCoy, A. J., Read, R. J. & Baker, D. (2007). *Nature*, **450**, 259–264. 10.1038/nature06249 PMC2504711 17934447

[bb37] Read, R. J. & McCoy, A. J. (2016). *Acta Cryst.* D**72**, 375–387. 10.1107/S2059798315013236 PMC4784668 26960124

[bb38] Remmert, M., Biegert, A., Hauser, A. & Söding, J. (2012). *Nat. Methods*, **9**, 173–175. 10.1038/nmeth.1818 22198341

[bb103] Richardson, L., Allen, B., Baldi, G., Beracochea, M., Bileschi, M. L., Burdett, T., Burgin, J., Caballero-Pérez, J., Cochrane, G., Colwell, L. J., Curtis, T., Escobar-Zepeda, A., Gurbich, T. A., Kale, V., Korobeynikov, A., Raj, S., Rogers, A. B., Sakharova, E., Sanchez, S., Wilkinson, D. J. & Finn, R. D. (2023). *Nucleic Acids Res.* **51**, D753–D759. 10.1093/nar/gkac1080 PMC9825492 36477304

[bb39] Rigden, D. J., Thomas, J. M. H., Simkovic, F., Simpkin, A., Winn, M. D., Mayans, O. & Keegan, R. M. (2018). *Acta Cryst.* D**74**, 183–193. 10.1107/S2059798318002310 PMC5947759 29533226

[bb40] Rodríguez, D. D., Grosse, C., Himmel, S., González, C., de Ilarduya, I. M., Becker, S., Sheldrick, G. M. & Usón, I. (2009). *Nat. Methods*, **6**, 651–653. 10.1038/nmeth.1365 19684596

[bb41] Ruiz-Serra, V., Pontes, C., Milanetti, E., Kryshtafovych, A., Lepore, R. & Valencia, A. (2021). *Proteins*, **89**, 1888–1900. 10.1002/prot.26248 PMC8616839 34595772

[bb43] Sammito, M., Meindl, K., de Ilarduya, I. M., Millán, C., Artola-Recolons, C., Hermoso, J. A. & Usón, I. (2014). *FEBS J.* **281**, 4029–4045. 10.1111/febs.12897 24976038

[bb42] Sammito, M., Millán, C., Rodríguez, D. D., de Ilarduya, I. M., Meindl, K., De Marino, I., Petrillo, G., Buey, R. M., de Pereda, J. M., Zeth, K., Sheldrick, G. M. & Usón, I. (2013). *Nat. Methods*, **10**, 1099–1101. 10.1038/nmeth.2644 24037245

[bb44] Sánchez Rodríguez, F., Simpkin, A. J., Davies, O. R., Keegan, R. M. & Rigden, D. J. (2020). *Acta Cryst.* D**76**, 962–970. 10.1107/S205979832001133X PMC7543657 33021498

[bb45] Simkovic, F., Thomas, J. M. H., Keegan, R. M., Winn, M. D., Mayans, O. & Rigden, D. J. (2016). *IUCrJ*, **3**, 259–270. 10.1107/S2052252516008113 PMC4937781 27437113

[bb47] Simpkin, A. J., Caballero, I., McNicholas, S., Stevenson, K., Jiménez, E., Sánchez Rodríguez, F., Fando, M., Uski, V., Ballard, C., Chojnowski, G., Lebedev, A., Krissinel, E., Usón, I., Rigden, D. J. & Keegan, R. M. (2023). *Acta Cryst.* D**79**, 806–819. 10.1107/S2059798323006289 PMC10478639 37594303

[bb49] Simpkin, A. J., Elliott, L. G., Stevenson, K., Krissinel, E., Rigden, D. & Keegan, R. M. (2022). *bioRxiv*, 2022.06.30.497974.

[bb48] Simpkin, A. J., Mesdaghi, S., Sánchez Rodríguez, F., Elliott, L., Murphy, D. L., Kryshtafovych, A., Keegan, R. M. & Rigden, D. J. (2023). *Proteins*, **91**, 1616–1635. 10.1002/prot.26593 PMC10792517 37746927

[bb46] Simpkin, A. J., Thomas, J. M. H., Simkovic, F., Keegan, R. M. & Rigden, D. J. (2019). *Acta Cryst.* D**75**, 1051–1062. 10.1107/S2059798319013962 PMC6889911 31793899

[bb60] Steinegger, M., Meier, M., Mirdita, M., Vöhringer, H., Haunsberger, S. J. & Söding, J. (2019). *BMC Bioinformatics*, **20**, 473. 10.1186/s12859-019-3019-7 PMC6744700 31521110

[bb50] Steinegger, M. & Söding, J. (2017). *Nat. Biotechnol.* **35**, 1026–1028. 10.1038/nbt.3988 29035372

[bb102] Suzek, B. E., Wang, Y., Huang, H., McGarvey, P. B., Wu, C. H. & UniProt Consortium (2015). *Bioinformatics*, **31**, 926–932. 10.1093/bioinformatics/btu739 PMC4375400 25398609

[bb51] Terwilliger, T. C., Afonine, P. V., Liebschner, D., Croll, T. I., McCoy, A. J., Oeffner, R. D., Williams, C. J., Poon, B. K., Richardson, J. S., Read, R. J. & Adams, P. D. (2023). *Acta Cryst.* D**79**, 234–244. 10.1107/S205979832300102X PMC9986801 36876433

[bb52] Thomas, J. M. H., Keegan, R. M., Bibby, J., Winn, M. D., Mayans, O. & Rigden, D. J. (2015). *IUCrJ*, **2**, 198–206. 10.1107/S2052252515002080 PMC4392414 25866657

[bb53] Thomas, J. M. H., Keegan, R. M., Rigden, D. J. & Davies, O. R. (2020). *Acta Cryst.* D**76**, 272–284. 10.1107/S2059798320000443 PMC7057219 32133991

[bb54] Wang, W., Peng, Z. & Yang, J. (2022). *Nat. Comput. Sci.* **2**, 804–814. 10.1038/s43588-022-00373-3 38177395

[bb55] Wells, J., Hawkins-Hooker, A., Bordin, N., Sillitoe, I., Paige, B. & Orengo, C. (2024). *Bioinformatics*, **40**, btae296. 10.1093/bioinformatics/btae296 PMC11256964 38718225

[bb56] Wu, T., Hou, J., Adhikari, B. & Cheng, J. (2020). *Bioinformatics*, **36**, 1091–1098. 10.1093/bioinformatics/btz679 PMC7703788 31504181

[bb57] Yamashita, K., Wojdyr, M., Long, F., Nicholls, R. A. & Murshudov, G. N. (2023). *Acta Cryst.* D**79**, 368–373. 10.1107/S2059798323002413 PMC10167671 37158197

[bb58] Zhang, L., Chen, C., Shen, T., Li, Y. & Sun, S. (2023). *arXiv*:2306.01824.

[bb59] Zhang, T., Ramakrishnan, R. & Livny, M. (1996). *SIGMOD Rec.* **25**, 103–114.

