# Supplementary material for: The success rate of processed predicted models in molecular replacement: implications for experimental phasing in the AlphaFold era
Source: Acta Crystallogr D Struct Biol. 2024 Oct 3;80(Pt 11):766–79. doi: 10.1107/S2059798324009380 (PMC11544426; doi:10.1107/S2059798324009380)
Supplement: Supplementary file 1 [file d-80-00766-sup1.pdf]

Acta Cryst D
STRUCTURAL BIOLOGY

Volume 80 (2024)

Supporting information for article:

The success rate of processed predicted models in molecular replacement: implications for experimental phasing in the *AlphaFold* era

Ronan M. Keegan, Adam J. Simpkin and Daniel J. Rigden

PDB IDS:

6st5, 7ej3, 7el5, 7eoz, 7ewf, 7fa1, 7fac, 7fax, 7fex, 7fgn, 7fgp, 7fh5, 7fia, 7fib, 7mch, 7o5i, 7o6t, 7o6u, 7o6v, 7o6w, 7p4a, 7pc1, 7pcv, 7pjr, 7pk2, 7pv5, 7pv7, 7pva, 7pwe, 7px1, 7pzt, 7q5y, 7qce, 7qda, 7qe1, 7qeh, 7qld, 7qlr, 7qrj, 7qrr, 7qsj, 7qwa, 7qwb, 7qwc, 7qwd, 7qwe, 7qx4, 7r0t, 7r0x, 7r1m, 7r6s, 7rdn, 7ris, 7rkb, 7rl3, 7roa, 7rt7, 7rxu, 7sfn, 7sfy, 7sie, 7smg, 7snr, 7sua, 7sz2, 7sz3, 7t5t, 7t69, 7t6a, 7tdr, 7tds, 7te2, 7th0, 7tlv, 7tlw, 7tom, 7tvl, 7twd, 7u01, 7u08, 7u2v, 7uav, 7uba, 7udi, 7uf8, 7ug8, 7ujb, 7ulo, 7ups, 7ur2, 7urp, 7usn, 7uv8, 7uvg, 7uwu, 7uyg, 7uzt, 7v0f, 7v1o, 7v2s, 7v3o, 7v52, 7v54, 7v56, 7v57, 7v7x, 7vbo, 7vch, 7vep, 7viv, 7vqk, 7vs2, 7vs6, 7vwv, 7vxt, 7vyu, 7w2o, 7w2q, 7w54, 7w63, 7w6y, 7w6z, 7w86, 7w91, 7wa9, 7wdt, 7wej, 7wh9, 7wjg, 7wjl, 7wlh, 7wmw, 7wmx, 7wmy, 7wmz, 7wn7, 7wrw, 7wuk, 7wup, 7wuw, 7wux, 7wuz, 7ww2, 7ww4, 7wwq, 7wwt, 7wwy, 7wx0, 7wx1, 7wzv, 7x0i, 7x0j, 7x0k, 7x0l, 7x0m, 7x0n, 7x0o, 7x15, 7x45, 7x6z, 7x7i, 7x8u, 7x9r, 7xbj, 7xcc, 7xds, 7xfp, 7xg9, 7xgt, 7xhz, 7xky, 7xmw, 7xn2, 7xp9, 7xpc, 7xpi, 7xpr, 7xrb, 7xre, 7xrx, 7xzf, 7y0y, 7y19, 7y3w, 7y56, 7y78, 7y79, 7y7o, 7y8u, 7yco, 7ydo, 7yfj, 7ygf, 7yhl, 7yik, 7yji, 7yjp, 7yjq, 7yjr, 7yjs, 7yjt, 7yk4, 7ykv, 7yle, 7ym5, 7ym7, 7ymo, 7yn1, 7ynx, 7ype, 7ypf, 7yr9, 7yt9, 7ytl, 7ytt, 7ytu, 7yuj, 7yv0, 7z3b, 7zbh, 7zgi, 7zhd, 7zhl, 7zju, 7zk1, 7znr, 7zns, 7ztw, 7zu3, 7zu8, 7zug, 7zv1, 7zyh, 8a14, 8a1i, 8a24, 8a2n, 8a30, 8a38, 8a82, 8aaj, 8ab2, 8abt, 8aew, 8aez, 8af9, 8ag9, 8ahd, 8ahz, 8aid, 8ajq, 8am4, 8asa, 8au6, 8auc, 8avz, 8ax2, 8ay2, 8b2e, 8b3e, 8b3w, 8b4l, 8b55, 8b73, 8b8d, 8bc5, 8bcx, 8bd1, 8bfh, 8bfi, 8bgt, 8bhd, 8bjw, 8bkd, 8bke, 8brp, 8bt6, 8bve, 8bvl, 8bvp, 8c3d, 8car, 8cgm, 8cnr, 8cpn, 8cwt, 8d0o, 8d3t, 8d89, 8da2, 8dc1, 8deh, 8df2, 8dfk, 8dop, 8dp6, 8dpk, 8dq2, 8dtn, 8dtq, 8dtu, 8dvq, 8dwz, 8ebf, 8ebg, 8efm, 8ehc, 8em5, 8emb, 8en9, 8ena, 8eo2, 8ep6, 8ewh, 8ezo, 8ezp, 8ezr, 8ezs, 8ezu, 8ezx, 8f00, 8f01, 8f03, 8f05, 8f06, 8f07, 8f0b, 8f3k, 8f7n, 8f8n, 8fbe, 8fby, 8fia, 8fnr, 8fns, 8fzz, 8g1n, 8g1y, 8g28, 8gbe, 8gj9, 8gjw, 8gjy, 8glb, 8gq9, 8gs1, 8gsx, 8gt9, 8gup, 8gxl, 8gy4, 8gy8, 8h0h, 8h0r, 8h1e, 8h1f, 8h1g, 8h3z, 8h8h, 8hav, 8haw, 8hbr, 8hd2, 8hdv, 8heh, 8hek, 8hhv, 8hja, 8hn2, 8hn3, 8hp8, 8hx3, 8i16, 8i2d, 8i3j, 8i59, 8i6h, 8i6z, 8i8y, 8ic1, 8iib, 8ilc, 8j1w, 8j1x, 8j67, 8j8p, 8j8q, 8j98, 8jj7, 8jpa, 8k1c, 8k1f, 8k1i, 8k5l, 8k76, 8ok4, 8opz, 8oq1, 8pfc, 8siu, 8smq, 8srz, 8tv0, 8u00, 8u01, 8u12

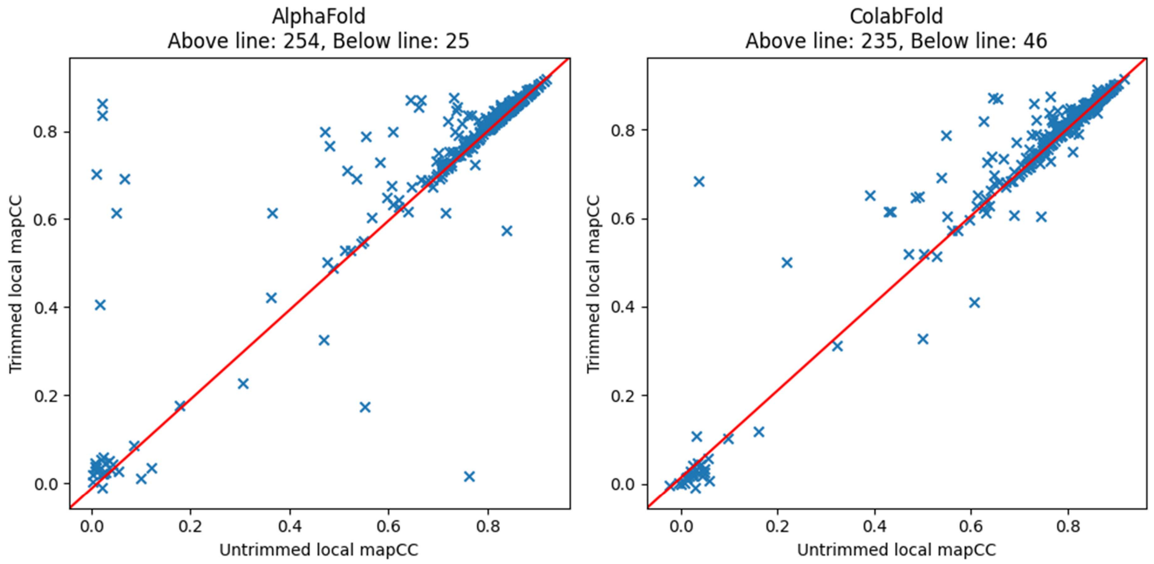

Figure S1 . A comparison of the local mapCC for AlphaFold2 and ColabFold models before and after trimming residues with a pLDDT <70. Points above the red line indicate that trimming has improved the mapCC and points below the red line indicate that trimming has decreased the mapCC.

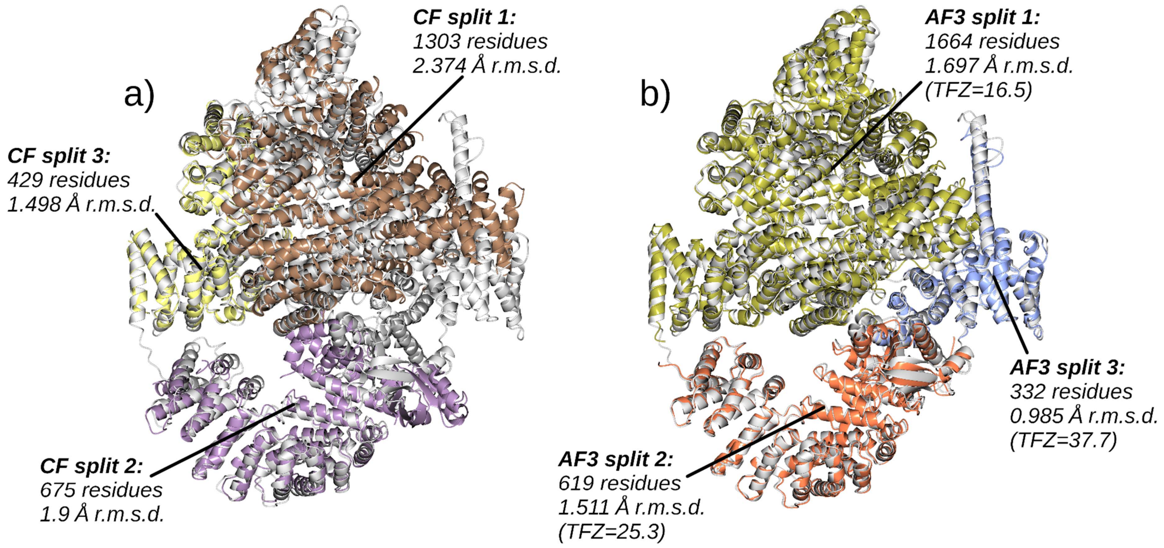

Figure S2 8bt6 (2.33Å, 1 copy in the asymmetric unit, spacegroup P212121). The CF (a) and AF3 (b) predictions split 3-ways by SnD and aligned to target (white) by Gesamt. Number of residues and r.m.s.d. to the target for each part are shown. Phaser was not successful in attempting to place the 3 search models created from the CF prediction but was able to place all 3 search models from the more accurate AF3 prediction with an LLG of 1794 (TFZ scores for each model are shown). Subsequent refinement of the

AF3 solution resulted in an R/Rfree of 0.25/0.32. Due to the size of the structure, we were unable to predict the target structure using AF2 as we lacked access to the computational resources needed. The AF3 prediction was generated using the Alphafold server (Abramson *et al.*, 2024).

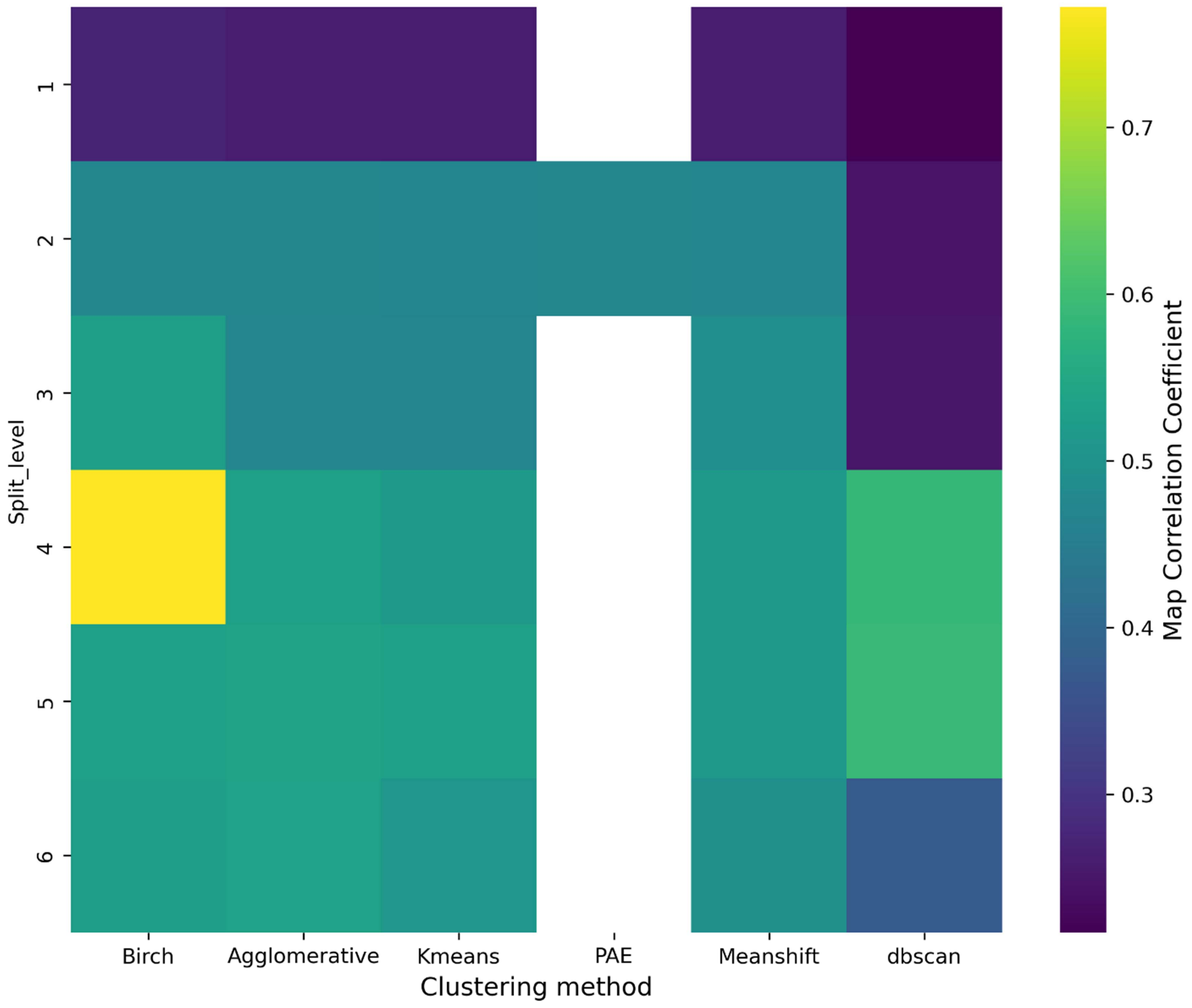

Figure S3 Comparison of MR results for 8ewh obtained with a range of clustering methods for splitting input structures applied to yield a range of numbers of splits. The dbscan method also removes outliers hence its difference to other methods. The heat map of results is colour coded according to the resulting map correlation coefficient.

Abramson, J., Adler, J., Dunger, J., Evans, R., Green, T., Pritzel, A., Ronneberger, O., Willmore, L., Ballard, A. J., Bambrick, J., Bodenstein, S. W., Evans, D. A., Hung, C.-C., O'Neill, M., Reiman, D., Tunyasuvunakool, K., Wu, Z., Žemgulytė, A., Arvaniti, E., Beattie, C., Bertolli, O., Bridgland, A., Cherepanov, A., Congreve, M., Cowen-Rivers, A. I., Cowie, A., Figurnov, M., Fuchs, F. B., Gladman, H., Jain, R., Khan, Y. A., Low, C. M. R., Perlin, K., Potapenko, A., Savy, P., Singh, S., Stecula, A., Thillaisundaram, A., Tong, C., Yakneen, S., Zhong, E. D., Zielinski, M., Žídek, A., Bapst, V., Kohli, P., Jaderberg, M., Hassabis, D. & Jumper, J. M. (2024). *Nature* **630**, 493–500.
